# Hospitalisation events in people with chronic kidney disease as a component of multimorbidity: parallel cohort studies in research and routine care settings

**DOI:** 10.1186/s12916-021-02147-6

**Published:** 2021-11-19

**Authors:** Michael K. Sullivan, Bhautesh Dinesh Jani, Alex McConnachie, Peter Hanlon, Philip McLoone, Barbara I. Nicholl, Juan-Jesus Carrero, Dorothea Nitsch, David McAllister, Frances S. Mair, Patrick B. Mark

**Affiliations:** 1 Institute of Cardiovascular and Medical Sciences, University of Glasgow, Glasgow, UK; 2 General Practice and Primary Care, Institute of Health and Wellbeing, University of Glasgow, Glasgow, UK; 3 Robertson Centre for Biostatistics, Institute of Health and Wellbeing, University of Glasgow, Glasgow, UK; 4 Public Health, Institute of Health and Wellbeing, University of Glasgow, Glasgow, UK; 5 Department of Medical Epidemiology and Biostatistics, Karolinska Institutet, Stockholm, Sweden; 6 Faculty of Epidemiology and Population Health, London School of Hygiene & Tropical Medicine, London, UK

**Keywords:** Chronic kidney disease, Multimorbidity, Comorbidity, Clinical epidemiology

## Abstract

**Background:**

Chronic kidney disease (CKD) typically co-exists with multimorbidity (presence of 2 or more long-term conditions: LTCs). The associations between CKD, multimorbidity and hospitalisation rates are not known. The aim of this study was to examine hospitalisation rates in people with multimorbidity with and without CKD. Amongst people with CKD, the aim was to identify risk factors for hospitalisation.

**Methods:**

Two cohorts were studied in parallel: UK Biobank (a prospective research study: 2006-2020) and Secure Anonymised Information Linkage Databank (SAIL: a routine care database, Wales, UK: 2011-2018). Adults were included if their kidney function was measured at baseline. Nine categories of participants were used: zero LTCs; one, two, three and four or more LTCs excluding CKD; and one, two, three and four or more LTCs including CKD. Emergency hospitalisation events were obtained from linked hospital records.

**Results:**

Amongst 469,339 UK Biobank participants, those without CKD had a median of 1 LTC and those with CKD had a median of 3 LTCs. Amongst 1,620,490 SAIL participants, those without CKD had a median of 1 LTC and those with CKD had a median of 5 LTCs. Compared to those with zero LTCs, participants with four or more LTCs (excluding CKD) had high event rates (rate ratios UK Biobank 4.95 (95% confidence interval 4.82–5.08)/SAIL 3.77 (3.71–3.82)) with higher rates if CKD was one of the LTCs (rate ratios UK Biobank 7.83 (7.42–8.25)/SAIL 9.92 (9.75–10.09)). Amongst people with CKD, risk factors for hospitalisation were advanced CKD, age over 60, multiple cardiometabolic LTCs, combined physical and mental LTCs and complex patterns of multimorbidity (LTCs in three or more body systems).

**Conclusions:**

People with multimorbidity have high rates of hospitalisation. Importantly, the rates are two to three times higher when CKD is one of the multimorbid conditions. Further research is needed into the mechanism underpinning this to inform strategies to prevent hospitalisation in this very high-risk group.

**Supplementary Information:**

The online version contains supplementary material available at 10.1186/s12916-021-02147-6.

## Background

Chronic kidney disease (CKD) is a global health problem and is closely linked to adverse outcomes [1]. Compared to those without CKD, people with CKD are more likely to be hospitalised [2], develop complications while in hospital [3] and be re-admitted [4]. They have frequent contacts with health care services: clinic visits, blood tests, procedures and in the case of advanced CKD, the need for dialysis and/or kidney transplantation. Unplanned hospitalisations are additional, undesirable events with heightened anxiety, particularly when admissions are via emergency services. CKD is typically accompanied by multimorbidity (the co-occurrence of two or more long-term conditions: LTCs), which may have caused CKD, developed as direct or indirect complications of CKD or are unrelated [5]. Multimorbidity has been identified by the medical community as a major challenge which should be made a research priority [6]. Polypharmacy and high treatment burden are frequently experienced by these people, which reduce their quality of life [7, 8]. Hospital admissions may be directly linked to CKD (fluid overload, vascular access surgery) or other illnesses which occur in excess in CKD (infections, cardiovascular events [2]). In addition to therapeutic intervention, people are exposed to the risk of the healthcare environment (e.g. nosocomial infection and isolation). However, there is a paucity of evidence about the implications of multimorbidity and CKD in those with mild to moderate CKD nor do we know the relationship between different types of LTCs and CKD [9].

In this study, we sought to fill this evidence gap and to examine the associations between CKD, multimorbidity and emergency admissions to hospital. We hypothesised that people with multimorbidity would have high rates of emergency hospitalisation and that the rates would be higher when CKD was one of the LTCs. We also hypothesised that amongst those with CKD, subgroups with proven susceptibilities to adverse outcomes would be high risk: those with advanced CKD [2], those living in socioeconomically deprived areas [10] and those with low body weight [11]. A 2021 National Institute for Health Research policy paper on multimorbidity states that improving our understanding of combinations of conditions, or clusters, may help develop strategies to prevent ill health [12]. We therefore explored the associations between hospitalisation and combinations of conditions which have been shown to be associated with increased risk of adverse outcomes: multiple cardiometabolic conditions [13] (i.e. heart failure, hypertension, coronary heart disease, peripheral vascular disease, atrial fibrillation, diabetes and stroke), complex LTCs [14] (three or more conditions from three or more body systems) and mixed physical and mental conditions [15].

Two different types of cohort were studied: first, a prospective cohort study was used because it has extensive clinical phenotyping and there is extensive published data demonstrating its utility for studying multimorbidity [16–20]. Second, because healthy volunteer bias can occur in research studies, a nationally representative primary care cohort generated from routine care records was used. This approach allowed us to confirm the generalisability of our findings to the general population.

## Methods

### Study design and setting

UK Biobank is a prospective research cohort with participants from England, Scotland and Wales. It enrolled volunteer participants aged 37 to 73 between 2006 and 2010, and they have been followed up since enrolment [21]. Individuals living within 25 miles of a UK Biobank assessment centre were invited to participate, and there was a 5% response rate. Each participant provided a detailed account of sociodemographic, lifestyle and medical information via a nurse-led interview and touchscreen questionnaire.

The Secure Anonymised Information Linkage Databank (SAIL) is a routine care database which holds anonymised primary care data for 79% of the population of Wales [22]. Our study included participants aged 18 to 108 with data after January 1, 2011. This date was chosen because recording of information before this date is incomplete [23]. Each participant has a random identifier which maintains confidentiality and ensures their identity stays the same if they relocate within Wales.

### Inclusion criteria

UK Biobank participants were included if their kidney function was measured at baseline. Adults over the age of 18 in SAIL were included if their kidney function was measured.

### Kidney function

The participants in each cohort were categorised into CKD (stages 3–5: estimated glomerular filtration rate: eGFR (using the CKD-EPI formula [24]) less than 60 ml/min/1.73m^2^) and non-CKD (eGFR greater than 60 ml/min/1.73m^2^). UK Biobank participants were assumed to be well and in a stable state of health when attending for assessment. Therefore, a single eGFR measured at the baseline assessment was used. Because results in SAIL are from routine care, we cannot assume a single eGFR result is during a stable state of health. To ensure reduced eGFRs reflect a chronic state, two results at least three months apart were used, in keeping with Kidney Disease: Improving Global Outcomes recommendations [25]. An alternative approach would have been to categorise participants without eGFR measurements as non-CKD [26]. This approach was included as a sensitivity analysis. Albuminuria was seldom recorded in SAIL, so it could not be used for the definition of CKD. Given albuminuria data were available in UK Biobank, we performed a sensitivity analysis by categorising participants with a urine albumin to creatinine ratio (uACR) greater than 30 mg/g as having CKD.

The UK Biobank biochemistry testing protocol has been detailed previously and calibrated analysers were used [27, 28]. Serum creatinine values for SAIL were taken from primary care data (Read codes, Additional File 1: Table S1). Given many different laboratories were used, creatinine values were multiplied by 0.95 to account for possible lack of calibration [29, 30].

### Primary analysis

Consistent with previous literature on LTCs in UK Biobank, 42 conditions additional to CKD were captured in both cohorts and limited to conditions present before cohort entry (Table 1) [16]. In UK Biobank, participants self-reported conditions and they entered the cohort on the date of baseline assessment. In SAIL, primary care Read codes (codes of clinical terms) were used to identify LTCs with prescription data confirming active treatment for some conditions (Additional File 1: Table S2) [31]. Participants in SAIL entered the cohort on the date of blood sampling (single sample for non-CKD and second, confirmatory sample for CKD). Participants were divided into nine categories. “Zero LTCs” was the reference category. Those without CKD were categorised as one LTC, two LTCs, three LTCs or four or more LTCs. Those with CKD were categorised as one LTC (i.e. CKD), two LTCs (i.e. CKD plus one other), three LTCs and four or more LTCs.

**Table 1:** Long-term conditions included

Hypertension	Peripheral vascular disease	Multiple sclerosis
Depression^m^	Atrial fibrillation	Parkinson’s disease
Asthma	Heart failure	Viral hepatitis
Coronary heart disease	Prostate disorders	Chronic liver disease
Diabetes mellitus	Glaucoma	Diverticular disease
Thyroid disease	Epilepsy	Osteoporosis
Connective tissue disease	Dementia^m^	Pernicious anaemia
Chronic obstructive pulmonary disease	Schizophrenia or bipolar affective disorder^m^	Endometriosis
Anxiety^m^	Psoriasis or eczema	Chronic fatigue syndrome
Irritable bowel syndrome	Inflammatory bowel disease	Polycystic ovarian syndrome
Cancer	Painful condition	Meniere’s disease
Alcohol problems^m^	Chronic sinusitis	Treated constipation
Psychoactive substance misuse^m^	Anorexia nervosa or bulimia^m^	Treated dyspepsia
Stroke or transient ischaemic attack	Bronchiectasis	Migraine

^m^Mental health conditions

### Types of condition

Conditions were categorised based on high-risk constellations of clinical disease groups [13–15]:

1. Cardiometabolic conditions
2. Complex pattern of conditions
  This was defined as the involvement of three or more body systems. Body systems were categorised using ICD-10 codes (infections, neoplasms, haematological, endocrine/metabolic, mental, neurological, ophthalmological, otological, circulatory, respiratory, gastrointestinal, dermatological, musculoskeletal, genitourinary and other).
3. Physical and mental conditions
  Mental health conditions are labelled in Table 1.

### Covariates

Ethnicity was categorised as White, Black, Asian, Mixed or Other. Socioeconomic status was quantified via deprivation scores and used as a continuous variable: Townsend [32] in UK Biobank and Welsh Index of Multiple Deprivation [33] (WIMD) in SAIL. Smoking status was categorised as never, current or previous. Body mass index (BMI), uACR and blood pressure were measured at baseline for UK Biobank. In SAIL, covariates were extracted using Read codes within 12 months of cohort entry (Additional File 1: Table S1).

### Outcomes

Emergency hospitalisation events, i.e. admissions to hospital, following the date of cohort entry were identified using linked hospital records. They were limited to emergencies by method of admission codes (Additional File 1: Table S3) [34]. Primary diagnoses were divided into systems of the body using Clinical Classification System categories [35]. Follow-up started on the date of baseline assessment for UK Biobank and the date of blood sampling for SAIL. Follow-up ended on 31 March 2020 for UK Biobank in Scotland and England; 28 February 2018 for UK Biobank in Wales; 31 May 2018 for SAIL; or on the date of death if this occurred earlier.

### Statistical analysis

Baseline characteristics were described for the CKD and non-CKD groups using medians and interquartile ranges for continuous variables and percentages for categorical variables. Differences in the distribution of these characteristics were tested using chi-squared tests for categorical variables and Kruskal-Wallis tests for continuous variables. Variables were also compared across LTC count categories.

Event rates were calculated by summing events for participants within each category over 100 years of follow-up and provided as per 100 person years. Rate ratios were calculated using negative binomial regression models. The linearity of the relationship between LTC counts and events was studied by plotting residuals against fitted values. Negative binomial models accounted for overdispersion [36]. Standard and zero-inflated models were compared to assess for excess zeroes using Vuong tests [37]. The log of duration of follow-up was included as an offset term. Adjustments were made for age, sex, smoking status and deprivation status as these variables have previously been linked to the risk of hospitalisation [30]. Given the risk of immortal time bias in SAIL for those with CKD, we built cox proportional hazards models using CKD diagnosis as a time-varying covariate [38]. Interactions between CKD status and LTC counts were tested by the addition of an interaction term to the models and the application of analysis of variance (ANOVA) tests between these and the standard models. Interactions were considered significant if *p* values were < 0.01.

Complete case analysis was deemed appropriate for UK Biobank as greater than 95% of the cohort had complete data. Multivariate imputation by chained equations [39] was performed in SAIL for smoking status and socioeconomic deprivation status with ten sets, each with ten iterations, assuming that these data were missing at random. Complete case sensitivity analysis was performed and these results were compared to the primary analysis.

### CKD participants: subgroup analysis

Amongst participants with CKD, the following subgroups were studied:

- Men and women
- CKD stages (3A, 3B and 4/5) [25]
- Age (< 50, 50–60, 60–70, 70–80* and > 80* years, *only available in SAIL)
- Deprivation quintiles (defined by distribution in the general cohort)
- BMI (< 25, 25–30 and > 30 kg/m^2^)

Event rates were compared to identify the subgroups most vulnerable to emergency hospitalisation. Rate ratios for each increase in LTC count were used to estimate the strength of association between increasing LTC count and hospitalisation.

### Type of condition

Amongst participants with CKD, the relationship between the type of LTC and hospitalisation was studied. The reference group was participants with zero or one LTC (excluding CKD as all participants in this part of the analysis had CKD). This was also performed for the non-CKD participants: the reference group was participants with zero or one LTC. This allowed us to compare the impact of type of LTC in people with and without CKD.

Statistical analyses were conducted using R version 4.0.3 (R Foundation for Statistical Computing, Vienna, AUT) with the tidyverse, MASS, pubh, survival, finalfit and forestplot packages.

## Results

### Participants

469,339 of 502,485 UK Biobank participants (93.4%) met the inclusion criteria and 10,767 (2.3%) of these had CKD. 1,620,490 of 2,611,238 adults in SAIL (62.1%) met the inclusion criteria and 173,388 (10.7%) of these had CKD. In SAIL, compared to those excluded from the analysis, those included tended to be older and had more LTCs (Additional File 1: Table S4).

### Baseline characteristics

#### UK Biobank

Compared to those without CKD, the participants with CKD had more LTCs, were older, were more likely to be ex-smokers and had higher BMI and higher systolic blood pressure (Table 2). Participants with more LTCs (whether CKD was included or excluded) were older, were more likely to be current or ex-smokers, lived in more deprived areas, with higher BMI, higher systolic blood pressure, higher uACR and lower eGFR (Additional File 1: Table S5).

**Table 2:** Baseline characteristics by chronic kidney disease (CKD) status

		UK Biobank			SAIL		
		No CKD *n* = 458 572 (97.7%)	CKD *n* = 10 767 (2.3%)	*P* value	No CKD *n* = 1 447 102 (89.3%)	CKD *n* = 173 388 (10.7%)	*P* value
Age (years)	Median (IQR)	58 (50 to 63)	64 (60 to 67)	< 0.001	50 (36 to 63)	79 (72 to 85)	< 0.001
Sex (%)	Female	248,751 (54.2)	5746 (53.4)	0.072	788,725 (54.5)	98,782 (57.0)	< 0.001
	Male	209,821 (45.8)	5021 (46.6)		658,377 (45.5)	74,606 (43.0)	
Ethnicity (%)	White	432,151 (94.7)	10,202 (95.3)	0.008	622,324 (94.5)	71,424 (98.6)	< 0.001
	Black	7173 (1.6)	128 (1.2)		6032 (0.9)	129 (0.2)	
	Asian	10,311 (2.3)	235 (2.2)		16,499 (2.5)	335 (0.7)	
	Mixed	2705 (0.6)	48 (0.4)		3806 (0.6)	105 (0.1)	
	Other	4085 (0.9)	96 (0.9)		10,061 (1.5)	222 (0.3)	
	Missing values	2205 (0.5%)			889,353 (54.9)		
Deprivation score^a^	Median (IQR)	− 2.2 (− 3.7 to 0.5)	− 2.0 (− 3.5 to 0.9)	< 0.001	18.0 (10.8 to 30.0)	17.6 (11.0 to 28.3)	< 0.001
	Missing values	577 (0.1 %)			302,952 (18.7%)		
Smoking status (%)	Never	250,304 (54.9)	5261 (49.3)	< 0.001	516,728 (48.4)	65,132 (47.4)	< 0.001
	Previous	157,573 (34.5)	4558 (42.7)		273,908 (25.7)	58,190 (42.4)	
	Current	48,402 (10.6)	862 (8.1)		276,545 (25.9)	13,962 (10.2)	
	Missing values	2379 (0.5%)			416,025 (25.7%)		
Body mass index (kg/m^2^)	Median (IQR)	27 (24 to 30)	29 (26 to 32)	< 0.001	29 (24 to 33)	29 (25 to 32)	0.394
	Missing values	1 873 (0.4%)			1 472 240 (90.9%)		
Systolic blood pressure (mmHg)	Median (IQR)	136 (125 to 150)	139 (127 to 152)	< 0.001	130 (120 to 141)	134 (122 to 144)	< 0.001
	Missing values	14 639 (3.1%)			287 529 (17.7%)		
Estimated glomerular filtration rate (ml/min/1.73m^2^)	Median (IQR)	93.1 (83.8 to 100.2)	54. 1 (48.1 to 57.6)	< 0.001	97.1 (85.4 to 109.4)	51.2 (42.9 to 56.2)	< 0.001
Urine albumin-creatinine ratio (mg/mmol)	Median (IQR)	0.0 (0.0 to 0.6)	0.4 (0.0 to 1.9)	< 0.001	0.9 (0.5 to 2.1)	1.8 (0.8 to 5.7)	< 0.001
	Missing values	13,406 (2.9%)			1,453,186 (89.7%)		
Long-term condition count (excluding CKD)	Median (IQR)	1 (0 to 2)	2 (1 to 3)	< 0.001	1 (0 to 2)	4 (2 to 5)	< 0.001

Interquartile range (IQR). *P* values: Kruskal-Wallis test for continuous variables and chi-squared tests for categorical variables

^a^Townsend score for UK Biobank: higher scores suggest higher levels of deprivation. Welsh Index of Multiple Deprivation Rank for SAIL: lower ranks suggest higher levels of deprivation

#### SAIL

Compared to those without CKD, participants with CKD had more LTCs, were older, there were proportionally more women and proportionally fewer non-White people and they were more likely to be ex-smokers with higher systolic blood pressure (Table 1). Participants with more LTCs (whether CKD was included or excluded) were older, were more likely to be ex-smokers, lived in less deprived areas, with higher BMI, higher systolic blood pressure, higher uACR and lower eGFR (Additional File 1: Table S6).

### Primary analysis

Median follow-up time in UK Biobank was 11.2 years (interquartile range (IQR) 10.5–11.9), and in SAIL, it was 8.0 years (IQR 6.5–8.2). There was a dose-response relationship between the number of LTCs and event rates in participants with and without CKD in both cohorts (Additional File 1: Table S7). Event rates were higher when CKD was included as an LTC, particularly in SAIL. The most common cause of hospitalisation was circulatory, especially in those with CKD (Additional File 1: Table S8).

A linear relationship was identified between LTC counts and log event rates in both cohorts whether CKD was included or not. Vuong tests demonstrated that standard models were superior to zero-inflated models (*P* < 0.001 in both cohorts).

**Fig. 1:**
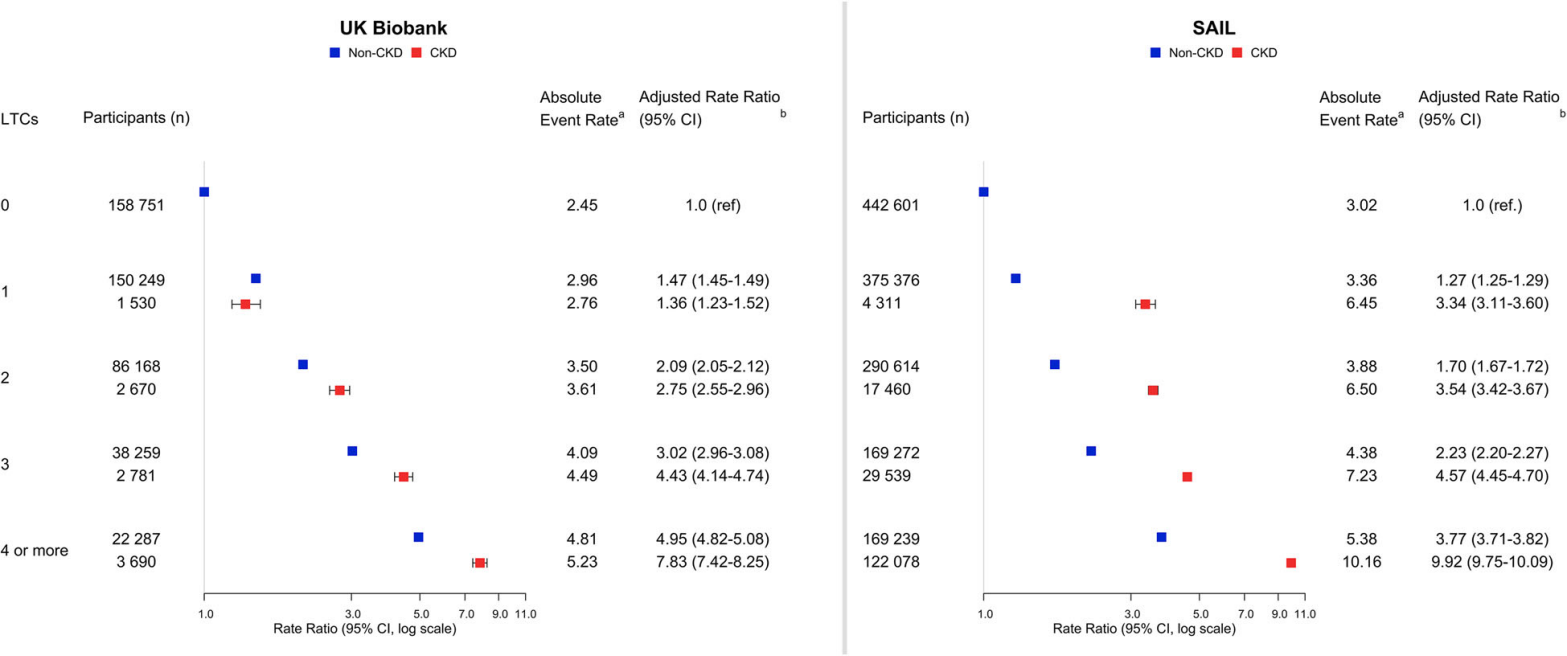
Hospitalisation events by chronic kidney disease (CKD) status and number of long-term conditions (LTCs). ^a^ indicates the following: events per 100 patient years. ^b^ indicates the following: adjusted for age, sex, deprivation status and smoking status. *P* values for all categories < 0.001

In both cohorts, event rates and rate ratios were highest in those with more LTCs (Fig. 1). For UK Biobank participants with one LTC, the rate ratios were similar for those with and without CKD. For SAIL participants with one LTC, the rate ratio was higher in those with CKD compared to those without CKD. With increasing numbers of LTCs in both cohorts, the rate ratios were higher, especially in those with CKD.

#### UK Biobank sensitivity analysis

Compared to the primary analysis, effect sizes were very similar when participants with albuminuria were categorised as having CKD (Additional File 1: Table S9).

#### SAIL sensitivity analyses

Compared to the primary analysis, effect sizes were higher for CKD participants when categorising those without biochemistry as non-CKD (Additional File 1: Table S10), but similar when using CKD diagnosis as a time-varying covariate (Additional File 1: Table S11) and in complete case analysis (Additional File 1: Table S12). There was evidence of multiplicative interactions between CKD status and the number of LTCs (*P* < 0.01 in both cohorts).

### CKD participants: subgroup analysis

#### Event rates in subgroups with CKD (Fig. 2)

Amongst those with CKD, event rates were similar for men and women. In both cohorts, participants over the age of 60 and participants with eGFRs less than 30 ml/min/1.73m^2^ had high event rates. In UK Biobank, event rates were highest in those living in the most deprived areas, but this was not the case in SAIL. In UK Biobank, participants with low BMIs had lower event rates than those with higher BMIs. The opposite trend was seen in SAIL, although proportionally few SAIL participants had BMI recorded and could be included in this part of the analysis.

**Fig. 2:**
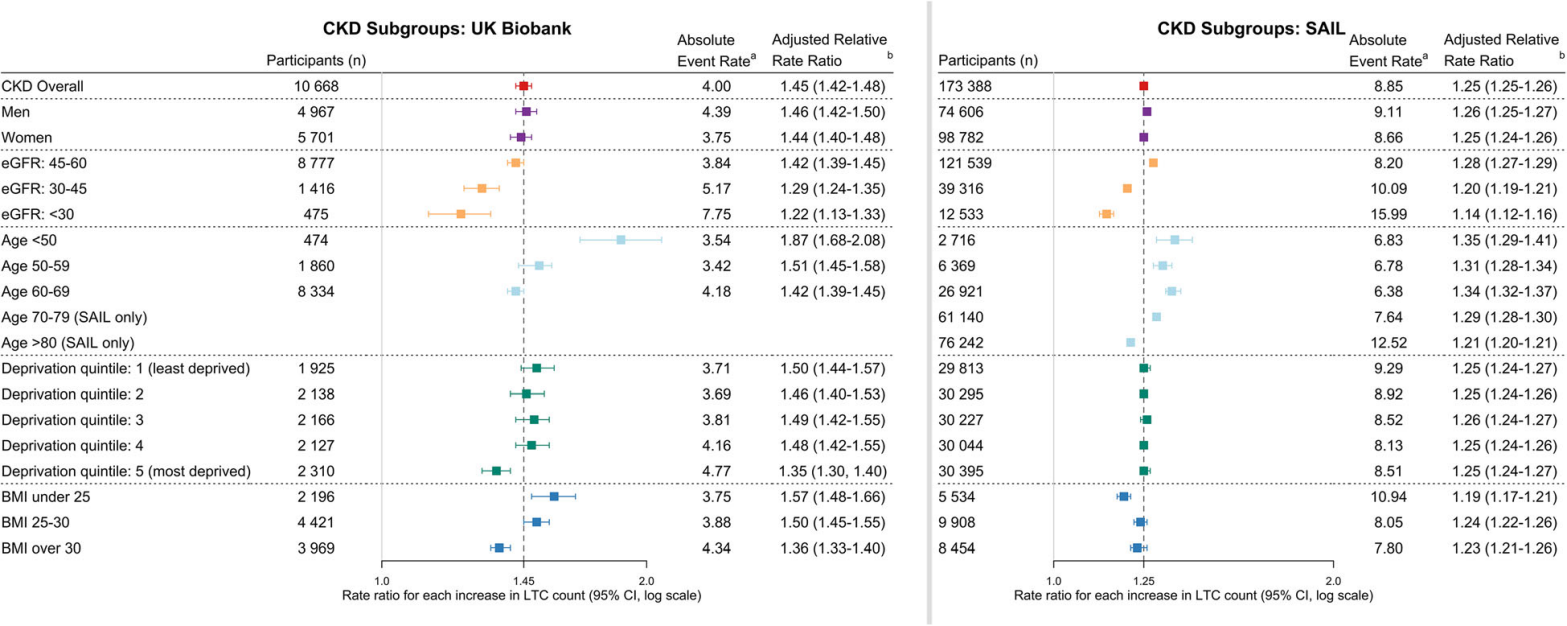
Risk of hospitalisation events in chronic kidney disease (CKD) participants with number of long-term conditions (LTCs) by sub-group. eGFR, estimate glomerular filtration rate. BMI, body mass index. ^a^ indicates the following: events per 100 patient years. ^b^ indicates the following: Adjusted for age, sex, deprivation status and smoking status. *P* values for all categories < 0.001

#### Rate ratios in subgroups with CKD (Fig. 2)

The importance of increasing LTC count as a risk factor was assessed via adjusted rate ratios for each increase in LTC. In both cohorts, adjusted rate ratios were similar for men and women and for participants from different deprivation quintiles. Adjusted rate ratios were higher in those under the age of 50 and those with eGFRs 45–60 ml/min/1.73m^2^ compared to older participants and those with lower eGFRs. In UK Biobank, the adjusted rate ratio was higher for those with BMI less than 25 kg/m^2^ than for those with higher BMI, but this trend was not seen in SAIL.

#### Type of condition (Fig. 3)

In both cohorts, participants with CKD and multiple cardiometabolic conditions were three to four times more likely to have events than those with CKD and zero or one LTC. Event rates for those with CKD and complex LTCs were approximately three times the rate of those with CKD and zero or one LTC. Participants with CKD, physical and mental health LTCs were approximately three times more likely to have events than those with CKD and zero or one LTC.

**Fig. 3:**
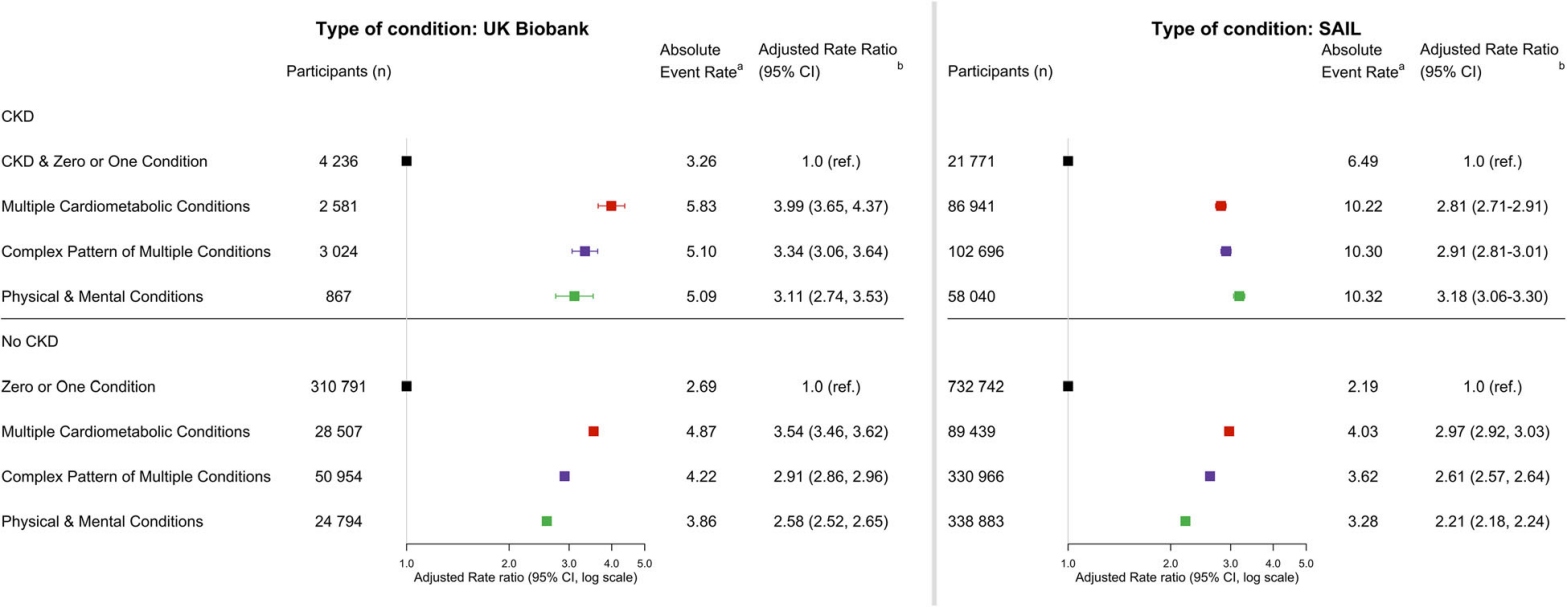
Hospitalisation events by type of condition. ^a^ indicates the following: events per 100 patient years. ^b^ indicates the following: Adjusted for age, sex, deprivation status and smoking status. *P* values for all categories < 0.001

In both cohorts, similar trends were seen for the non-CKD participants, but with lower effect sizes compared to the CKD participants (except for cardiometabolic LTCs in SAIL). In SAIL, combined physical and mental conditions was a more significant risk factor in CKD participants (adjusted rate ratio 3.18: 3.06–3.30) compared to non-CKD participants (adjusted rate ratio 2.21: 2.18–2.24).

## Discussion

We have studied emergency hospitalisations in a combined 2.1 million individuals from a prospective research study and a routine care database. Those with more LTCs had high rates of emergency hospitalisation and the risk was substantially increased by two to threefold in those with CKD (depending on the cohort). We also showed that the type of LTCs was important: those with CKD plus multiple cardiometabolic conditions, complex LTCs and physical and mental health LTCs were at heightened risk of hospitalisation.

Previous studies have identified a relationship between reduced eGFR and hospitalisation [2, 40]. Others have examined cohorts of patients with CKD and demonstrated that patients with LTCs are at high risk of hospitalisation [41]. What has not been studied before is how CKD relates to hospitalisation compared to, or in combination with, other LTCs. We have demonstrated that CKD is not equivalent to other LTCs as part of a multimorbidity count, but rather that individuals with CKD as one of the LTCs are particularly vulnerable to hospitalisation. In our study, people with CKD were frequently admitted with cardiovascular problems. This vulnerability to cardiovascular problems amongst people with CKD is well known, and it undoubtedly contributed to the overall high rates of hospitalisation in our study. The use of sodium-glucose cotransporter-2 inhibitors may prevent a proportion of these admissions in future [42]. Because numerous multimorbidity measures exist, researchers are encouraged to use a measure which suits their purpose [43]. Our study supports this message, emphasising that CKD is a critical condition in these measures and its significance should not be overlooked.

Amongst those with CKD, we found low eGFR and advanced age to be associated with high hospitalisation rates. However, regression analyses showed there was a disproportionately strong association between the number of LTCs and hospitalisation in those under the age of 50. We had hypothesised that the link between LTCs and hospitalisation would be strongest in those with more advanced CKD. We were surprised to find that the association between the number of LTCs and hospitalisation was less strong than the same association in those with mild to moderate CKD. It may be that because people with advanced CKD are primarily elderly, most of them have multiple LTCs and they have such a high baseline rate of hospitalisation, the influence of additional LTCs is attenuated. A previous study in 530,771 Canadians with CKD studied the link between the type of LTC and adverse outcomes [41]. As in our study, they found associations between LTCs and hospitalisation. In their study, this relationship was not unique to concordant LTCs, with associations also seen for discordant and mental health conditions. We have meaningfully extended this subject area by finding that certain combinations of LTCs were associated with heightened risk of hospitalisation (cardiometabolic, complex and physical/mental LTCs).

These findings are important for patients, carers, healthcare professionals and policy makers. As people with CKD and LTCs are known to be high-risk, clinicians caring for them should provide targeted monitoring. This does not mean monitoring of blood tests in isolation, as people with CKD and multimorbidity should have regular, thorough reviews of their clinical status, medications and preferences. CKD is common in the general population and although asymptomatic in the early stages, knowing which people have CKD may be helpful for healthcare planning. Combined physical and mental health conditions were a risk factor for hospitalisation in our study. Although we cannot assume that mental health support would prevent hospitalisations, people with mental and physical conditions are in need of psychological support [44], which has been proven to reduce depression and improve self-management [45].

Alternative strategies to hospitalisation exist, with improvements in quality of life for patients and cost reductions for healthcare systems [46]. Safe and effective care can be provided for outpatient management of illnesses such as pneumonia [47]. Alternatively, some people may not wish to be hospitalised and “Hospital at Home” services [48] and/or anticipatory care planning [49] may be better for some people. Clinicians should be mindful of these strategies when seeing people with multiple health conditions, and they should discuss the options during routine appointments, so their patients know what alternatives to emergency admission exist. Care models like these are not appropriate for all people or all illnesses, but when they are used, they can be beneficial for patients and less costly for healthcare systems. Structured interventions are, however, not always successful [50], and incentivisation may be necessary to reduce admissions [51].

Our study has several strengths. Using two large cohorts, we have expanded from a research setting with healthy volunteer bias [52] to a routine care database to confirm the generalisability of our findings in the general population. The use of linked healthcare records with universal coverage in the UK ensures we have identified most hospitalisations [53]. UK Biobank has been used extensively to study risk factors for health outcomes, but it sometimes draws criticism for not being representative of the general population [52]. Although event rates were higher in SAIL and the general population were older with more LTCs, the trends identified in UK Biobank were similar in SAIL.

Our study has some limitations. Although we have adjusted for age in our regression models, there is still a possibility of residual confounding. Some risk factors are undoubtedly on the causal pathways to our exposures and our outcome (e.g. obesity, alcohol use). It has not been possible in this study to unravel the complex relationships between all risk factors, exposures and the outcome. The relative lack of ethnic diversity in these particular UK cohorts means that the study should be replicated in other contexts. The eGFR equation we used (CKD-EPI) incorporates ethnicity, and there is not yet consensus in the medical community about whether this is appropriate [54]. LTCs in UK Biobank were self-reported and this risks the introduction of error. Although self-report may be less accurate for some conditions such as heart failure [55], it has been found to be a valid approach [56, 57]. CKD status, LTCs and covariates were only taken at baseline and we have not taken into account changes during follow-up. Data about severity of conditions would have been informative (particularly for some conditions such as heart failure and chronic obstructive pulmonary disease), but this was not available. Regardless, it would have been difficult to synthesise such information for 42 conditions. We employed counts of conditions rather than an index which assigns scores to conditions associated with greater morbidity. The evidence regarding whether simple counts or weighted measures are preferable is mixed [58] with some systematic reviews concluding that both are equally effective at predicting most outcomes [59]. A meta-review of six systematic reviews on this topic concluded there is a lack of a clear consensus, and it suggested selection of measures should depend on the purpose of any given study [60]. Our finding that CKD is linked to a heightened risk of hospitalisation may be transferable to other specific conditions, but we have not repeated it for each condition. We excluded 37.9% of the SAIL population without biochemistry data, who tended to be younger with fewer additional LTCs than those included. Sensitivity analysis showed that the difference in hospitalisation rates between non-CKD and CKD groups widened when participants without biochemistry were categorised as non-CKD. SAIL participants were lost to follow-up if they move away from Wales, which means some hospitalisation events may have not been recorded. The rates of missing data were high for some variables in SAIL. Multiple imputation was used, with similar results obtained in complete case analysis.

## Conclusions

People with increasing multimorbidity count are therefore at high risk of emergency hospitalisation, and the rates are two to threefold higher when CKD is present. People with CKD at heightened risk of hospitalisation should be targeted by research aimed at addressing emergency hospital admissions.

## Supplementary Information

**Additional file 1: Table S1.** Read codes. **Table S2**. Long-term conditions considered and read code definitions used in Secure Anonymised Information Linkage Databank. mMental health condition. **Table S3**. Method of admission codes to identify emergency admissions. HES, Hospital Episode Statistics, SMR, Scottish Morbidity Record, PEDW, Patient Episode Database for Wales, A&E Accident & Emergency, GP, General Practitioner, NHS National Health Service. **Table S4**. Baseline characteristics in SAIL by study inclusion. IQR, interquartile range, WIMD Welsh Index of Multiple Deprivation. **Table S5**. Baseline characteristics by chronic kidney disease (CKD) status and number of long-term conditions (LTCs) for UK Biobank. IQR, interquartile range. **Table S6**. Baseline characteristics by chronic kidney disease (CKD) status and number of long-term conditions (LTCs) for SAIL. IQR, interquartile range. **Table S7**. Hospitalisation Events by Chronic Kidney Disease (CKD) status and number of long-term conditions (LTCs). *Adjusted for age, sex, deprivation status and smoking status. **Table S8**. Causes of Hospitalisation. CKD, chronic kidney disease. **Table S9**. UK Biobank Sensitivity analysis. Analysis categorising participants with albuminuria as Chronic Kidney Disease (CKD). Hospitalisation Events by CKD status and number of long-term conditions (LTCs). *Adjusted for age, sex, Welsh Index of Multiple Deprivation and smoking status. *P* values <.001 for all comparisons. **Table S10**. SAIL Sensitivity analysis 1. Analysis including participants without biochemistry categorised as no Chronic Kidney Disease (CKD). Hospitalisation Events by CKD status and number of long-term conditions (LTCs). * Adjusted for age, sex, Welsh Index of Multiple Deprivation and smoking status. *P* values <.001 for all comparisons. **Table S11**. SAIL Sensitivity analysis 2. Analysis using chronic kidney disease (CKD) diagnosis as a time-varying covariate. Hospitalisation Events by CKD status and number of long-term conditions (LTCs). * Adjusted for age, sex, Welsh Index of Multiple Deprivation and smoking status. *P* values <.001 for all comparisons. **Table S12**. SAIL Sensitivity analysis 3. Complete case analysis: Hospitalisation Events by Chronic Kidney Disease (CKD) status and number of long-term conditions (LTCs). * Adjusted for age, sex, Welsh Index of Multiple Deprivation and smoking status. *P* values <.001 for all comparisons.

## Data Availability

The data that support the findings of this study are available from UK Biobank and SAIL, subject to successful registration and application process. Further details can be found at ukbiobank.ac.uk and saildatabank.com.

## Abbreviations

ANOVA: Analysis of variance
BMI: Body mass index
CKD: Chronic kidney disease
eGFR: Estimated glomerular filtration rate
IQR: Interquartile range
LTCs: Long-term conditions
SAIL: Secure Anonymised Information Linkage Databank
uACR: Urine albumin to creatinine ratio
WIMD: Welsh Index of Multiple Deprivation
