# Supplementary material for: Hospitalisation events in people with chronic kidney disease as a component of multimorbidity: parallel cohort studies in research and routine care settings
Source: BMC Med. 2021 Nov 19;19:278. doi: 10.1186/s12916-021-02147-6 (PMC8603496; doi:10.1186/s12916-021-02147-6)
Supplement: Supplementary file 1 — Additional file 1: Table S1. Read codes. Table S2. Long-term conditions considered and read code definitions used in Secure Anonymised Information Linkage Databank. mMental health condition. Table S3. Method of admission codes to identify emergency admissions. HES, Hospital Episode Statistics, SMR, Scottish Morbidity Record, PEDW, Patient Episode Database for Wales, A&E Accident & Emergency, GP, General Practitioner, NHS National Health Service. Table S4. Baseline characteristics in SAIL by study inclusion. IQR, interquartile range, WIMD Welsh Index of Multiple Deprivation. Table S5. Baseline characteristics by chronic kidney disease (CKD) status and number of long-term conditions (LTCs) for UK Biobank. IQR, interquartile range. Table S6. Baseline characteristics by chronic kidney disease (CKD) status and number of long-term conditions (LTCs) for SAIL. IQR, interquartile range. Table S7. Hospitalisation Events by Chronic Kidney Disease (CKD) status and number of long-term conditions (LTCs). *Adjusted for age, sex, deprivation status and smoking status. Table S8. Causes of Hospitalisation. CKD, chronic kidney disease. Table S9. UK Biobank Sensitivity analysis. Analysis categorising participants with albuminuria as Chronic Kidney Disease (CKD). Hospitalisation Events by CKD status and number of long-term conditions (LTCs). *Adjusted for age, sex, Welsh Index of Multiple Deprivation and smoking status. P values <.001 for all comparisons. Table S10. SAIL Sensitivity analysis 1. Analysis including participants without biochemistry categorised as no Chronic Kidney Disease (CKD). Hospitalisation Events by CKD status and number of long-term conditions (LTCs). * Adjusted for age, sex, Welsh Index of Multiple Deprivation and smoking status. P values <.001 for all comparisons. Table S11. SAIL Sensitivity analysis 2. Analysis using chronic kidney disease (CKD) diagnosis as a time-varying covariate. Hospitalisation Events by CKD status and number of long-term conditions (LTCs). [file 12916_2021_2147_MOESM1_ESM.docx]

| **Variable** | **Read Code(s)** |
| --- | --- |
| **Laboratory** | |
| Serum Creatinine | 44J33, 44J30, 44J3Z, 44J3., EMISREQ\|44J3, 44JF., 44J32, 44JC., 44JD., 44J31, 4Q40. |
| Total Cholesterol | 44P.., 44OE., 44PH., 44PJ., 44PK., 662a., 6879., 44PF., 44PZ., 44P9. |
| Urine albumin:creatinine ratio | 46TC., 44J7. |
| **Smoking** | |
| Never-Smoker | 1371., 137L. |
| Ex-Smoker | 1377., 1378., 1379., 137A., 137B., 137F., 137j., 137K., 137l., 137N., 137O., 137S., 9km.. |
| Smoker | 137.., 1372., 1373., 1374., 1375., 1376., 137a., 137H., 137J., 137P., 137Q., 137R., 137X., 137Y., 137Z., 9ko.. |
| **Body Measurements** | |
| Weight | 22A.. |
| Height | 229.. |
| Systolic blood pressure | 2469., 246N., 246Q., 246S., 246b., 246d., 246e., 246W., 246Y., 246l., 246.. |

**Table S1. Read codes**

| Long-term condition | Condition Read Codes | Additional Requirements |
| --- | --- | --- |
| Hypertension | 662G., 662O., 662P., 662q., 662r., 8B26., 8BL0., 8CR4., F4042, F4213, F4504, G2..., G20.., G200., G201., G202., G203., G20z., G21.., G210., G2100, G2101, G210z, G211., G2110, G2111, G211z, G21z., G21z0, G21z1, G21zz, G22.., G220., G221., G222., G22z., G23.., G230., G231., G232., G233., G234., G23z., G24.., G240., G2400, G240z, G241., G2410, G241z, G244., G24z., G24z0, G24z1, G24zz, G2y.., G2z.., G672., Gyu2., Gyu20, Gyu21 |  |
| Depression^m^ | 1B17., 1B1U., 1BT.., 2257, 62T1., 66590, 6G00., 8CAa., 8HHq., 9H90., 9H91., 9H92., 9HA0., E0013, E002., E0021, E002z, E0043, E02y3, E112., E1120, E1121, E1122, E1123, E1124, E1125, E1126, E112z, E113., E1130, E1131, E1132, E1133, E1134, E1135, E1136, E1137, E113z, E118., E11y2, E11z2, E130., E135., E204., E2003, E290., E290z, E291., E2B.., E2B0., E2B1., Eu204, Eu251, Eu32., Eu320, Eu321, Eu322, Eu323, Eu324, Eu325, Eu326, Eu327, Eu328, Eu329, Eu32A, Eu32y, Eu32z, Eu33., Eu330, Eu331, Eu332, Eu333, Eu334, Eu33y, Eu33z, Eu341, Eu412, Eu920, ZN120, ZN121, ZN123, ZN124, ZN125 | OR Four or more prescriptions for antidepressants issued per year. Medication read Codes:  d8... , daD.. , daD1. , daD2. , du61. , du6z. , da9.. , da91. , da92. , da93. , da94. , da95. , da96. , da97. , da98. , da99. , da9A. , da9z. , gde3. , gde4. , gdew. , gdex. , daC.. , daC1. , daC2. , daC3. , daC4. , daC5. , daC6. , daC7. , daC8. , daC9. , daCA. , da4.. , da41. , da42. , da43. , da44. , da45. , da46. , da47. , da48. , da49. , da4A. , da4B. , da4C. , da3.. , da31. , da32. , da33. , da34. , d82.. , d821. , d822. , d82y. , d82z. , d83.. , d831. , d83z. , d7b.. , d7b1. , d7b2. , d7b3. , d7b4. , d7b5. , d7b6. , d7b7. , d7b8. , d7b9. , daB.. , daB1. , daB2. , daB3. , daB4. , daB5. , daB6. , daB7. , daB8. , daBy. , daBz. , d85.. , d851. , d852. , d853. , d854. , da8.. , da81. , da82. , da83. , da84. , da85. , da86. , da6.. , da61. , da62. , da63. , da64. , da65. , da66. , da67. , da68. , d81.. , d811. , d81z. , daA.. , daA1. , daA2. , da5.. , da51. , da52. , da53. , da54. , d84.. , d841. , d84z. , d7e.. , d7e1. , d7e2. , d7e3. , d7e4. , d7e5. , d7e6. , d7e7. , d7ew. , d7ex. , d7ez. , da2.. , da21. , da22. , da23. , da24. , da2y. , da2z. , da7.. , da71. , da72. , da73. , da74. , da75. , da76. , da77. , da78. , da79. , da7a. , da7A. , da7b. , da7B. , da7c. , da7C. , da7d. , da7D. , da7e. , da7E. , da7f. , da7F. , da7g. , da7G. , da7h. , da7H. , da7i. , da7I. , da7j. , da7J. , da7k. , da7K. , da7l. , da7L. , da7m. , da7M. , da7n. , da7N. , da7o. , da7O. , da7p. , da7P. , da7q. , da7Q. , da7r. , da7R. , da7s. , da7S. , da7T. , da7U. , da7V. , da7W. , da7X. , da7Y. , da7Z. , d7g.. , d7g1. , d7gz. , daE.. , daE2. , daE4. , daE6. |
| Asthma | 173A., 173c., 173d., 1780, 1O2.., 663d., 663e., 6.63E+02, 6.63E+03, 663f., 663h., 663j., 663m., 663n., 663N., 663N0, 663N1, 663N2, 663O., 663O0, 663p., 663P., 663q., 663Q., 663r., 663s., 663t., 663u., 663U., 663v., 663V., 663P., 663q., 663r., 663u., 663v., 663V0, 663V1, 663V2, 663V3, 663w., 663W., 663x., 663y., 66Y5., 66Y9., 66YA., 66YC., 66YE., 66YJ., 66YK., 66YP., 66YQ., 66YR., 8793, 8794, 8795, 8796, 8797, 8798, 8B3j., 8CR0., 8791, 9hA.., H3120, H33.., H330., H3300, H3301, H330z, H331., H3310, H3311, H331z, H332., H333., H334., H33z., H33z0, H33z1, H33z2, H33zz, H35y6, H35y7, H47y0 | AND Four or more prescriptions for asthma medication issued per year. Medication read codes:  c1... , c3... , c4... , cA... , cl... , c34.. , c341. , c342. , c41.. , c411. , c412. , c413. , c414. , c415. , c416. , c417. , c418. , c41a. , c41A. , c41b. , c41B. , c41c. , c41C. , c41d. , c41e. , c41f. , c41g. , c41h. , c41i. , c41j. , c41k. , c41m. , c1B.. , c1B1. , c1B2. , c1B3. , c1B4. , c61.. , c611. , c612. , c613. , c614. , c616. , c617. , c619. , c61a. , c61A. , c61b. , c61B. , c61c. , c61C. , c61d. , c61D. , c61e. , c61E. , c61f. , c61F. , c61g. , c61G. , c61h. , c61H. , c61i. , c61j. , c61J. , c61k. , c61K. , c61l. , c61L. , c61m. , c61M. , c61n. , c61N. , c61O. , c61p. , c61P. , c61q. , c61Q. , c61r. , c61R. , c61s. , c61S. , c61t. , c61T. , c61u. , c61v. , c61V. , c61w. , c61W. , c61x. , c61X. , c61y. , c61Y. , c61z. , c61Z. , c66.. , c661. , c662. , c663. , c664. , c665. , c666. , c667. , c668. , c669. , c66a. , c66A. , c66b. , c66B. , c66c. , c66C. , c66d. , c66D. , c66e. , c66E. , c66f. , c66F. , c66g. , c66G. , c66h. , c66H. , c66I. , c66J. , c66K. , c66L. , c66M. , c66N. , c66P. , c66Q. , c66R. , c66S. , c66T. , c66U. , c66V. , c66W. , c66X. , c66Y. , c66Z. , c63.. , c631. , c63z. , c64.. , c641. , c642. , c643. , c644. , c645. , c647. , c648. , c649. , c64a. , c64A. , c64b. , c64B. , c64c. , c64C. , c64d. , c64D. , c64e. , c64E. , c64F. , c64g. , c64G. , c64h. , c64H. , c64i. , c64I. , c64j. , c64J. , c64k. , c64K. , c64l. , c64L. , c64m. , c64M. , c64n. , c64N. , c64o. , c64p. , c64u. , c64v. , c64w. , c64x. , c64y. , c64z. , c42.. , c421. , c422. , c423. , c424. , c42w. , c42x. , c42y. , c42z. , c69.. , c691. , c692. , c69y. , c69z. , c71.. , c711. , c712. , c713. , c714. , c715. , c716. , c717. , c718. , c719. , c71a. , c71b. , c71c. , c71d. , c71e. , c71f. , c71g. , c71h. , c71i. , c71j. , c71k. , c22.. , c221. , c222. , c223. , c224. , c225. , c226. , c227. , c21.. , c213. , c216. , c15.. , c151. , c152. , c153. , c154. , c15y. , c15z. , c51A. , c51B. , c51i. , c51v. , c51w. , c51x. , c65.. , c651. , c652. , c653. , c654. , c655. , c656. , c657. , c658. , c659. , c65a. , c65A. , c65b. , c65B. , c65c. , c65C. , c65d. , c65D. , c65e. , c65E. , c65f. , c65F. , c65g. , c65G. , c65H. , c65I. , c65J. , c65K. , c65L. , c65M. , c65N. , c65O. , c65P. , c65Q. , c65R. , c65S. , c65T. , c65U. , c65V. , c65W. , c65X. , c65Y. , c65Z. , c1C.. , c1C1. , c1C2. , c1C3. , c1C4. , c1C5. , c1C6. , c1C7. , c1C8. , c1Cy. , c1Cz. , c1c.. , c1c1. , c1c2. , c1c3. , c1cx. , c1cy. , c1cz. , c67.. , c671. , c672. , c673. , c67x. , c67y. , c67z. , c6A.. , c6A1. , c6Az. , o323. , o324. , c1b.. , c1b1. , c1b2. , c1b3. , c1b4. , c31.. , c311. , c312. , c313. , c314. , c315. , c316. , c317. , c318. , c319. , c31A. , c31B. , c31C. , c31D. , c31E. , c31F. , c31G. , c31t. , c31u. , c31v. , c31w. , c31x. , c31y. , c31z. , c23.. , c231. , c23z. , c24.. , c243. , c245. , c246. , c24x. , c24y. , c24z. , c75.. , c752. , c68.. , c681. , c682. , c683. , c684. , cA1.. , cA11. , cA12. , cA13. , cA14. , cA15. , cA16. , cA1y. , cA1z. , c74.. , c741. , c742. , c743. , c744. , c745. , c746. , c747. , c1d.. , c1d2. , ck1.. , ck11. , ck12. , ck13. , ck14. , ck15. , ck16. , c251. , c252. , c254. , c255. , c25v. , c25w. , c25y. , c25z. , c32.. , c321. , c322. , c323. , c324. , c16.. , c161. , c162. , c163. , c164. , c16w. , c16x. , c16y. , c16z. , c17.. , c173. , c17y. , c18.. , c181. , c182. , c183. , c184. , c18y. , c18z. , cl1.. , cl11. , cl1z. , c11.. , c111. , c112. , c113. , c114. , c115. , c116. , c118. , c119. , c11a. , c11b. , c11c. , c11d. , c11D. , c11e. , c11f. , c11g. , c11h. , c11i. , c11j. , c11k. , c11m. , c11n. , c11o. , c11p. , c11q. , c11v. , c11x. , c11y. , c11z. , c12.. , c121. , c122. , c123. , c124. , c125. , c126. , c12w. , c12x. , c12y. , c12z. , c13.. , c131. , c132. , c133. , c134. , c135. , c136. , c137. , c139. , c13a. , c13A. , c13B. , c13C. , c13d. , c13D. , c13e. , c13E. , c13f. , c13F. , c13g. , c13G. , c13h. , c13H. , c13i. , c13I. , c13j. , c13J. , c13K. , c13l. , c13L. , c13m. , c13M. , c13n. , c13N. , c13o. , c13O. , c13p. , c13P. , c13q. , c13Q. , c13r. , c13R. , c13S. , c13T. , c13U. , c13v. , c13V. , c13w. , c13W. , c13x. , c13X. , c13y. , c13Y. , c13z. , c13Z. , c1E.. , c1E1. , c1E2. , c1E3. , c1E4. , c1E5. , c1E6. , c1E7. , c1E8. , c1E9. , c1EA. , c1EB. , c1EC. , c1ED. , c1EE. , c51C. , c51D. , c51E. , c51F. , c51G. , c51H. , c531. , c722. , c723. , c72y. , c72z. , c19.. , c191. , c192. , c193. , c194. , c195. , c196. , c197. , c198. , c199. , c19A. , c19B. , c19z. , c1D.. , c1D1. , c1D2. , c1D3. , c1D4. , c1D5. , c1D6. , c1Du. , c1Dv. , c1Dw. , c1Dx. , c1Dy. , c1Dz. , c14.. , c141. , c142. , c143. , c144. , c145. , c146. , c147. , c148. , c149. , c14a. , c14b. , c14c. , c14e. , c14f. , c14g. , c14h. , c14i. , c14j. , c14k. , c14r. , c14s. , c14t. , c14u. , c14v. , c14w. , c14x. , c14y. , c14z. , c43.. , c431. , c432. , c433. , c434. , c435. , c436. , c437. , c438. , c439. , c43a. , c43A. , c43b. , c43B. , c43c. , c43d. , c43e. , c43f. , c43g. , c43h. , c43i. , c43j. , c43k. , c43m. , c43n. , c43o. , c43p. , c43q. , c43r. , c43s. , c43t. , c43u. , c43v. , c43w. , c43x. , c43y. , c43z. , c51t. , c51u. , c33.. , c331. , c332. , c333. , c33x. , c33y. , c33z. , c1a.. , c1a1. , c1a2. , c1a3. , c1a4. , c1a5. , cA2.. , cA21. , cA22. |
| Coronary Heart Disease | 14AL., G3..., G30B., G31.., G310., G3110, G312., G31y., G31y0, G31y1, G31y2, G31y3, G31yz, G32.., G33z., G33z0, G33z1, G33z2, G33z3, G33z4, G33z5, G33z6, G33z7, G33zz, G34.., G340., G3400, G3401, G34.., G340., G3400, G3401, G3412, G342., G343., G344., G34y., G34y0, G34y1, G34yz, G34z., G34z0, G35.., G350., G351., G353., G35X., G36.., G360., G361., G362., G363., G364., G365., G366., G38.., G380., G381., G382., G383., G384., G38z., G3y.., G3z.., G501., G5y2., Gyu3., Gyu31, Gyu32, Gyu33, Gyu35, Gyu36 |  |
| Diabetes Mellitus | 13AB., 13AC., 13B1., 1434, 14F4., 2BBF., 2BBk., 2BBL., 2BBl., 2BBo., 2BBP., 2BBQ., 2BBR., 2BBr., 2BBS., 2BBT., 2BBV., 2BBW., 2BBX., 2G510, 2G5A., 2G5B., 2G5C., 2G5E., 2G5F., 2G5G., 2G5H., 2G5I., 2G5J., 2G5K., 2G5L., 2G5V., 2G5W., 3881, 3882, 3883, 42c.., 42c0., 42c1., 42c2., 42W.., 42W1., 42W2., 42W3., 42WZ., 44V3., 66A.., 66A1., 66A2., 66A3., 66A4., 66A5., 66A6., 66A7., 66A70, 66A71, 66A8., 66A9., 66Aa., 66AA., 66Ab., 66AB., 66Ac., 66AC., 66Ad., 66AD., 66Ae., 66Af., 66Ag., 66Ah., 66AH., 66AH0, 66Ai., 66AI., 66Aj., 66AJ., 66AJ0, 66AJ1, 66AJ2, 66AJ3, 66AJz, 66Ak., 66AK., 66Al., 66AL., 66Am., 66AM., 66An., 66AN., 66Ao., 66Ap., 66AP., 66Aq., 66AQ., 66AR., 66AS., 66AT., 66AU., 66AV., 66AW., 66AX., 66AY., 66AZ., 6761, 7L198, 8A12., 8A13., 8A17., 8A19., 8B3l., 8CA41, 8CP2., 8CR2., 8CS0., C10.., C100., C1000, C1001, C100z, C101., C1010, C1011, C101y, C101z, C102., C1020, C1021, C102z, C103., C1030, C1031, C103y, C103z, C104., C1040, C1041, C104y, C104z, C105., C1050, C1051, C105y, C105z, C106., C1060, C1061, C106y, C106z, C107., C1070, C1071, C1072, C1073, C1074, C107y, C107z, C108., C1080, C1081, C1082, C1083, C1084, C1085, C1086, C1087, C1088, C1089, C108A, C108B, C108C, C108D, C108E, C108F, C108G, C108H, C108J, C108y, C108z, C109., C1090, C1091, C1092, C1093, C1094, C1095, C1096, C1097, C1099, C109A, C109B, C109C, C109D, C109E, C109F, C109G, C109H, C109J, C109K, C10A., C10A0, C10A1, C10A2, C10A3, C10A4, C10A5, C10A6, C10A7, C10AW, C10AX, C10B., C10B0, C10C., C10D., C10E., C10E0, C10E1, C10E2, C10E3, C10E4, C10E5, C10E6, C10E7, C10E8, C10E9, C10EA, C10EB, C10EC, C10ED, C10EE, C10EF, C10EG, C10EH, C10EJ, C10EK, C10EL, C10EM, C10EN, C10EP, C10EQ, C10ER, C10F., C10F0, C10F1, C10F2, C10F3, C10F4, C10F5, C10F6, C10F7, C10F9, C10FA, C10FB, C10FC, C10FD, C10FE, C10FF, C10FG, C10FH, C10FJ, C10FK, C10FL, C10FM, C10FN, C10FP, C10FQ, C10FR, C10FS, C10G., C10G0, C10H., C10H0, C10M., C10M0, C10N., C10N0, C10N1, C10y., C10y0, C10y1, C10yy, C10yz, C10z., C10z0, C10z1, C10zy, C10zz, C11y0, Cyu2., Cyu20, Cyu21, Cyu22, Cyu23, F1711, F3450, F35z0, F372., F3720, F3721, F3722, F3813, F3y0., F420., F4200, F4201, F4202, F4203, F4204, F4205, F4206, F4207, F4208, F420z, F4407, F4640, G73y0, K01x1, L1805, L1806, L1807, L180X, Lyu29, M0372, M2710, M2711, M2712, N0300, N0301, Q441., R0542, R0543 |  |
| Thyroid Disease | 22H2., 22H3., 22H4., 66B.., 66B1., 66B2., 66B3., 66B4., 66B5., 66B7., 66B8., 66B9., 66BB., 66BZ., 8CR5., C0..., C00.., C000., C00z., C01.., C010., C011., C01z., C03.., C03y., C03y0, C03y1, C03z., C04.., C040., C041., C0410, C041z, C042., C043., C0430, C0431, C0432, C043z, C044., C047., C04y., C04z., C04z0, C04z1, C05.., C050., C0500, C0501, C0502, C050z, C051., C052., C053., C054., C05y., C05y4, C05z., C06.., C060., C061., C062., C063., C0630, C0631, C063z, C06y., C06y0, C06y1, C06yz, C06z., C0A.., C0A0., C0A1., C0A2., C0A3., C0A4., C0A5., C0AX., C1343, C3A.., Cyu1., Cyu10, Cyu11, Cyu12, Cyu14, Cyu15, Cyu4J, F11x5, F1441, F3814, L1810, PK251, Q4337 |  |
| Connective Tissue Disease | 14G1., 669.., 6691, 6692, 6693, 6697, 6699, 669Z., 66c0., AD61., C34.., C340., C341., C3410, C3411, C341z, C342., C343., C344., C345., C34y., C34y0, C34y1, C34y2, C34y3, C34y4, C34y5, C34yz, C34z., C394., F371., F3710, F3711, F3712, F371z, F3961, F3963, F3964, F3966, F3967, F4A32, G5573, G5yA., G5y8., G75.., G750., G751., G7510, G751z, G752., G7520, G752z, G753., G754., G755., G7550, G7551, G7552, G755z, G756., G7560, G7561, G756z, G757., G758., G759., G75X., G75z., H570., H572., H57y1, H57y3, H57y4, K01x3, K01x4, K0B40, M1601, M1602, N00.., N000., N0000, N0001, N0002, N0003, N0004, N0005, N000z, N001., N0010, N0011, N0012, N002., N003., N0030, N0031, N003X, N004., N005., N00y., N00y0, N00y1, N00z., N012., N0120, N0121, N0122, N0123, N0124, N0125, N0126, N0127, N012x, N012y, N012z, N02.., N020., N0200, N0201, N0202, N0203, N0204, N0205, N0206, N0207, N020x, N020y, N020z, N021., N0210, N0211, N0212, N0213, N0214, N0215, N0216, N0217, N021x, N021y, N021z, N022., N0220, N0221, N0222, N0223, N0224, N0225, N0226, N0227, N022x, N022y, N022z, N023., N0230, N0231, N0232, N0233, N0234, N0235, N0236, N0237, N0238, N023x, N023y, N023z, N024., N02y., N02y0, N02y1, N02y2, N02y3, N02y4, N02y5, N02y6, N02y7, N02y8, N02yx, N02yy, N02yz, N02z., N02z0, N02z1, N02z2, N02z3, N02z4, N02z5, N02z6, N02z7, N02z8, N02z9, N02zA, N02zB, N02zC, N02zD, N02zE, N02zF, N02zG, N02zH, N02zJ, N02zK, N02zL, N02zM, N02zN, N02zP, N02zQ, N02zR, N02zS, N02zT, N02zx, N02zy, N02zz, N03.., N030., N0302, N031., N032., N036., N03x., N03x0, N03x1, N03x2, N03x3, N03x4, N03x5, N03x6, N03x7, N03x8, N03x9, N03xA, N03xB, N03xC, N03xD, N03xE, N03xF, N03xG, N03xH, N03xJ, N03xK, N03y., N03z., N0237, N0238, N023x, N023y, N023z, N024., N02y., N02y0, N02y1, N02y2, N02y3, N02y4, N02y5, N02y6, N02y7, N02y8, N02yx, N02yy, N02yz, N02z., N02z0, N02z1, N02z2, N02z3, N02z4, N02z5, N02z6, N02z7, N02z8, N02z9, N02zA, N02zB, N02zC, N02zD, N02zE, N02zF, N02zG, N02zH, N02zJ, N02zK, N02zL, N02zM, N02zN, N02zP, N02zQ, N02zR, N02zS, N02zT, N02zx, N02zy, N02zz, N03.., N030., N0302, N031., N032., N036., N03x., N03x0, N03x1, N03x2, N03x3, N03x4, N03x5, N03x6, N03x7, N03x8, N03x9, N03xA, N03xB, N03xC, N03xD, N03xE, N03xF, N03xG, N03xH, N03xJ, N03xK, N03y., N03z., N023x, N04.., N040., N0400, N0401, N0402, N0403, N0404, N0405, N0406, N0407, N0408, N0409, N040A, N040B, N040C, N040D, N040E, N040F, N040G, N040H, N040J, N040K, N040L, N040M, N040N, N040P, N040Q, N040R, N040S, N040T, N041., N042., N0420, N0421, N0422, N042z, N043., N0430, N0431, N0432, N0433, N043z, N0451, N0452, N0453, N0454, N0455, N0456, N047., N04X., N04y., N04y2, N04y3, N04yz, N04z., N0505, N060., N0600, N0601, N0602, N0603, N0604, N0605, N0606, N0607, N0608, N0609, N060z, N062., N0620, N0621, N0622, N0623, N0624, N0625, N0626, N0627, N0628, N0629, N062z, N063., N0630, N0631, N0632, N0633, N0634, N0635, N0636, N0637, N0638, N0639, N063z, N065., N0650, N0651, N0652, N0653, N0654, N0655, N0656, N0657, N0658, N0659, N065A, N065z, N068., N069., N06y., N06y0, N06y1, N06y2, N06y3, N06y4, N06y5, N06y6, N06y7, N06y8, N06y9, N06yz, N06zA, N06zB, N06zz, N090W, N092., N0920, N0921, N0922, N0923, N0924, N0925, N0926, N0927, N0928, N0929, N092A, N092B, N092C, N092D, N092E, N092F, N092G, N092H, N092J, N092K, N092L, N092M, N092N, N092P, N092Q, N092R, N092S, N092T, N092U, N092V, N092z, N093., N0930, N0931, N0932, N0933, N0934, N0935, N0936, N0937, N0938, N0939, N093z, N0y.., N0z.., N20.., N200., N2208, N235., N2432, Nyu00, Nyu1., Nyu10, Nyu11, Nyu12, Nyu13, Nyu14, Nyu15, Nyu16, Nyu17, Nyu18, Nyu19, Nyu1A, Nyu1B, Nyu1C, Nyu1D, Nyu1E, Nyu1F, Nyu1G, Nyu4., Nyu40, Nyu41, Nyu42, Nyu43, Nyu44, Nyu45, Nyu46, Nyu47, Nyu48, Nyu49, Nyu4A, Nyu4B, Nyu4C, Nyu4D, Nyu4E, Nyu4F |  |
| Chronic Obstructive Pulmonary Disease | 66YB., 66Yd., 66YD., 66Ye., 66Yf., 66Yg., 66Yh., 66Yi., 66YI., 66YL., 66YM., 66YS., 66YT., 8CR1., H3..., H30.., H300., H301., H302., H30z., H31.., H310., H3100, H3101, H310z, H311., H3110, H3111, H311z, H312., H3120, H3121, H3122, H312z, H313., H31y., H31y0, H31y1, H31yz, H31z., H32.., H320., H3200, H3201, H3202, H3203, H320z, H321., H322., H32y., H32y0, H32y1, H32y2, H32yz, H32z., H36.., H37.., H38.., H39.., H3y.., H3y0., H3y1., H3z.., H4640, H4641, Hyu3., Hyu30, Hyu31 |  |
| Anxiety^m^ | 285.., 286.., 1466, 1B13., 225J., 8G94., E2..., E20.., E200., E2000, E2001, E2002, E2003, E2004, E2005, E200z, E201., E2010, E2011, E2012, E2013, E2014, E2015, E2016, E2017, E2018, E2019, E201A, E201B, E201C, E201z, E202., E2020, E2021, E2022, E2023, E2024, E2025, E2026, E2027, E2028, E2029, E202A, E202B, E202C, E202D, E202E, E202z, E203., E2030, E2031, E203z, E204., E205., E206., E207., E20y., E20y0, E20y1, E20y2, E20y3, E20yz, E20z., E21.., E210., E211., E2110, E2111, E2112, E2113, E211z, E212., E2120, E2121, E2122, E212z, E213., E214., E2140, E2141, E214z, E215., E2150, E2151, E2152, E2153, E215z, E216., E217., E21y., E21y0, E21y1, E21y2, E21y3, E21y4, E21y5, E21y6, E21y7, E21yz, E21z., E26.., E260., E2600, E2601, E260z, E261., E2610, E2611, E2612, E2613, E2614, E2615, E261z, E262., E2620, E2621, E2622, E2623, E262z, E263., E2630, E263z, E264., E2640, E2642, E2643, E2644, E2645, E264z, E265., E2650, E2651, E2652, E2653, E265z, E266., E267., E26y., E26y0, E26yz, E26z., E278., E2780, E2781, E2782, E278z, E28.., E280., E281., E282., E283., E2830, E2831, E283z, E284., E28z., E29.., E2900, E2920, E2921, E2922, E2923, E2924, E2925, E292y, E292z, E293., E2930, E2931, E2932, E293z, E294., E29y., E29y0, E29y1, E29y2, E29y3, E29y4, E29y5, E29yz, E29z., E292., Eu054, Eu4.., Eu40., Eu400, Eu401, Eu402, Eu403, Eu40y, Eu40z, Eu41., Eu410, Eu411, Eu412, Eu413, Eu41y, Eu41z, Eu42., Eu420, Eu421, Eu422, Eu42y, Eu42z, Eu43., Eu430, Eu431, Eu432, Eu43y, Eu43z, Eu44., Eu440, Eu441, Eu442, Eu443, Eu444, Eu445, Eu446, Eu447, Eu44y, Eu44z, Eu45., Eu450, Eu451, Eu452, Eu453, Eu454, Eu455, Eu45y, Eu45z, Eu46., Eu460, Eu461, Eu46y, Eu46z, Eu930, Eu931, Eu932, M240E, Eu45., Eu450, Eu451, Eu455, Eu45y, Eu45z, Eu46., ZN114, ZS7C7 | OR Four or more prescriptions for anxiolytics issued per year. Medication read codes:  d2... , d22.. , d221. , d222. , d22y. , d22z. , d23.. , d231. , d232. , d23y. , d23z. , d2f.. , d2f1. , d2f2. , d2f3. , d2f4. , d2f5. , d24.. , d241. , d242. , d243. , d244. , d245. , d246. , d247. , d248. , d249. , d24a. , d24b. , d24c. , d24d. , d24e. , d24f. , d24g. , d24h. , d24i. , d24j. , d26.. , d261. , d262. , d263. , d264. , d265. , d266. , d267. , d268. , d21.. , d211. , d212. , d213. , d214. , d215. , d216. , d217. , d218. , d219. , d21a. , d21A. , d21b. , d21B. , d21c. , d21C. , d21d. , d21D. , d21e. , d21E. , d21f. , d21F. , d21g. , d21G. , d21h. , d21J. , d21k. , d21l. , d21m. , d21n. , d21o. , d21p. , d21q. , d21r. , d21s. , d21t. , d21u. , d21v. , d21y. , d21z. , o53.. , o531. , o532. , o533. , o534. , o535. , d28.. , d281. , d282. , d283. , d284. , d285. , d28x. , d28y. , d28z. , d29.. , d291. , d292. , d29y. , d29z. , d2a.. , d2a1. , d2a2. , d2a3. , d2a4. , d2a5. , d2a6. , d2a7. , d2az. , do41. , d2b.. , d2b1. , d2b2. , d2by. , d2bz. , d2c.. , d2c1. , d2c2. , d2c3. , d2c4. , d2c5. , d2c6. , d2d.. , d2d1. , d2d2. , d2d3. , d2d4. , d2d5. , d2d6. , d2d7. , d27.. , d271. , d272. , d27y. , d27z. , d2e.. , d2e1. , d2ez. |
| Irritable Bowel Syndrome | 14CF., J521., J5210, J529., J52y., J52yz, J52z., Jyu53 |  |
| Cancer | 1O0.., 7G03K, A7886, A788W, A7898, B0..., B00.., B000., B0000, B0001, B000z, B001., B0010, B0011, B001z, B002., B0020, B0021, B0022, B0023, B002z, B003., B0030, B0031, B0032, B0033, B003z, B004., B0040, B0041, B0042, B0043, B004z, B005., B006., B007., B00y., B00z., B00z0, B00z1, B00zz, B01.., B010., B0100, B010z, B011., B0110, B0111, B011z, B012., B013., B0130, B0131, B013z, B014., B015., B016., B017., B01y., B01z., B02.., B020., B021., B022., B023., B02y., B02z., B03.., B030., B031., B03y., B03z., B04.., B040., B041., B042., B04y., B04z., B05.., B050., B051., B0510, B0511, B0512, B0513, B051z, B052., B053., B054., B055., B0550, B0551, B055z, B056., B05y., B05z., B06.., B060., B0600, B0601, B0602, B060z, B061., B062., B0620, B0621, B0622, B0623, B062z, B063., B064., B0640, B0641, B064z, B065., B066., B067., B06y., B06y0, B06yz, B06z., B07.., B070., B071., B0710, B0711, B071z, B072., B0720, B0721, B072z, B073., B0730, B0731, B0732, B073z, B074., B07y., B07z., B08.., B080., B081., B082., B083., B084., B08y., B08z., B0z.., B0z0., B0z1., B0z2., B0zy., B0zz., B1..., B10.., B100., B101., B102., B103., B104., B105., B106., B107., B10y., B10z., B11.., B110., B1100, B1101, B110z, B111., B1110, B1111, B111z, B112., B113., B114., B115., B116., B117., B118., B119., B11y., B11y0, B11y1, B11yz, B11z., B12.., B120., B121., B122., B123., B124., B12y., B12z., B13.., B130., B131., B132., B133., B134., B135., B136., B137., B138., B139., B13y., B13z., B14.., B140., B141., B142., B1420, B143., B14y., B14z., B15.., B150., B1500, B1501, B1502, B1503, B150z, B151., B1510, B1511, B1512, B1513, B1514, B151z, B152., B153., B15z., B16.., B160., B161., B1610, B1611, B1612, B1613, B161z, B162., B163., B16y., B16z., B17.., B170., B171., B172., B173., B174., B175., B176., B17y., B17y0, B17yz, B17z., B18.., B180., B1800, B1801, B1802, B180z, B181., B182., B18y., B18y0, B18y1, B18y2, B18y3, B18y4, B18y5, B18y6, B18y7, B18yz, B18z., B1z.., B1z0., B1z1., B1z10, B1z11, B1z1z, B1z2., B1zy., B1zz., B2..., B20.., B200., B2000, B2001, B2002, B2003, B200z, B201., B2010, B2011, B2012, B2013, B201z, B202., B203., B204., B205., B206., B20y., B20z., B21.., B210., B211., B212., B213., B2130, B2131, B2132, B2133, B213z, B214., B215., B21y., B21z., B22.., B220., B2200, B2201, B220z, B221., B2210, B2211, B221z, B222., B2220, B2221, B222z, B223., B2230, B2231, B223z, B224., B2240, B2241, B224z, B225., B226., B22y., B22z., B23.., B230., B231., B232., B23y., B23z., B24.., B240., B241., B2410, B2411, B2412, B2413, B2414, B241z, B242., B243., B24X., B24y., B24z., B25.., B26.., B2z.., B2z0., B2zy., B2zz., B3..., B30.., B300., B3000, B3001, B3002, B3003, B3004, B3005, B3006, B3007, B3008, B3009, B300A, B300B, B300C, B300z, B301., B302., B3020, B3021, B3022, B302z, B303., B3030, B3031, B3032, B3033, B3034, B3035, B303z, B304., B3040, B3041, B3042, B3043, B3044, B304z, B305., B3050, B3051, B3052, B3053, B3054, B3055, B3056, B3057, B3058, B3059, B305A, B305B, B305C, B305D, B305z, B306., B3060, B3061, B3062, B3063, B3064, B3065, B306z, B307., B3070, B3071, B3072, B307z, B308., B3080, B3081, B3082, B3083, B3084, B3085, B3086, B3087, B3088, B3089, B308A, B308B, B308C, B308D, B308z, B309., B30W., B30X., B30z., B30z0, B31.., B310., B3100, B3101, B3102, B3103, B3104, B3105, B310z, B311., B3110, B3111, B3112, B3113, B3114, B3115, B311z, B312., B3120, B3121, B3122, B3123, B3124, B3125, B3126, B312z, B313., B3130, B3131, B3132, B3133, B313z, B314., B3140, B3141, B314z, B315., B3150, B3151, B3152, B3153, B315z, B316., B317., B31y., B31z., B31z0, B32.., B320., B321., B322., B3220, B3221, B322z, B323., B3230, B3231, B3232, B3233, B3234, B3235, B323z, B324., B3240, B3241, B324z, B325., B3250, B3251, B3252, B3253, B3254, B3255, B3256, B3257, B3258, B325z, B326., B3260, B3261, B3262, B3263, B3264, B3265, B326z, B327., B3270, B3271, B3272, B3273, B3274, B3275, B3276, B3277, B3278, B3279, B327z, B32y., B32y0, B32z., B33.., B330., B331., B3310, B3311, B3312, B332., B3320, B3321, B3322, B332z, B333., B3330, B3331, B3332, B3333, B3334, B3335, B333z, B334., B3340, B3341, B334z, B335., B3350, B3351, B3352, B3353, B3354, B3355, B3356, B3357, B3358, B3359, B335A, B335z, B336., B3360, B3361, B3362, B3363, B3364, B3365, B336z, B337., B3370, B3371, B3372, B3373, B3374, B3375, B3376, B3377, B3378, B3379, B337z, B338., B339., B33X., B33y., B33z., B33z0, B33z1, B34.., B340., B3400, B3401, B340z, B341., B342., B343., B344., B345., B346., B347., B34y., B34y0, B34yz, B34z., B35.., B350., B3500, B3501, B350z, B35z., B35z0, B35zz, B3y.., B3z.., B4..., B40.., B41.., B410., B4100, B4101, B410z, B411., B412., B41y., B41y0, B41y1, B41yz, B41z., B42.., B420., B43.., B430., B4300, B4301, B4302, B4303, B430z, B431., B4310, B431z, B432., B43y., B43z., B44.., B440., B441., B442., B443., B444., B44y., B44z., B45.., B450., B4500, B4501, B450z, B451., B4510, B451z, B452., B453., B454., B45X., B45y., B45y0, B45z., B46.., B47.., B470., B4700, B4701, B4702, B4703, B470z, B471., B4710, B4711, B471z, B47z., B48.., B480., B481., B482., B483., B484., B485., B486., B487., B48y., B48y0, B48y1, B48y2, B48yz, B48z., B49.., B490., B491., B492., B493., B494., B495., B496., B497., B49y., B49y0, B49z., B4A.., B4A0., B4A00, B4A1., B4A10, B4A11, B4A1z, B4A2., B4A3., B4A4., B4Ay., B4Ay0, B4Az., B4y.., B4z.., B5..., B50.., B500., B5000, B5001, B5002, B5003, B500z, B501., B5010, B5011, B501z, B502., B503., B504., B505., B506., B507., B5070, B5071, B507z, B508., B50y., B50z., B51.., B510., B5100, B5101, B5102, B5103, B5104, B5105, B510z, B511., B512., B5120, B5121, B512z, B513., B514., B515., B5150, B5151, B515z, B516., B517., B5170, B5171, B5172, B5173, B517z, B51y., B51y0, B51y1, B51y2, B51yz, B51z., B52.., B520., B5200, B5201, B5202, B520z, B521., B5210, B5211, B5212, B521z, B522., B523., B5230, B5231, B5232, B523z, B524., B5240, B5241, B5242, B5243, B5244, B5245, B5246, B524W, B524X, B525., B52W., B52X., B52y., B52z., B53.., B54.., B540., B5400, B5401, B540z, B541., B542., B5420, B5421, B542z, B543., B544., B545., B5450, B5451, B5452, B545z, B546., B54X., B54y., B54z., B55.., B550., B5500, B5501, B5502, B5503, B5504, B5505, B550z, B551., B5510, B5511, B5512, B551z, B552., B553., B5530, B5531, B5532, B553z, B554., B555., B55y., B55y0, B55y1, B55y2, B55yz, B55z., B56.., B560., B5600, B5601, B5602, B5603, B5604, B5605, B5606, B5607, B5608, B5609, B560z, B561., B5610, B5611, B5612, B5613, B5614, B5615, B5616, B5617, B5618, B5619, B561z, B562., B5620, B5621, B5622, B5623, B5624, B562z, B563., B5630, B5631, B5632, B5633, B563z, B564., B5640, B5641, B5642, B564z, B565., B5650, B5651, B5652, B5653, B5654, B565z, B56y., B56z., B57.., B570., B571., B572., B573., B574., B5740, B5741, B5742, B574z, B575., B5750, B5751, B575z, B576., B5760, B5761, B5762, B576z, B577., B57y., B57z., B58.., B580., B581., B5810, B5811, B5812, B581z, B582., B5820, B5821, B5822, B5823, B5824, B5825, B5826, B582z, B583., B5830, B5831, B5832, B583z, B584., B585., B5850, B586., B587., B58y., B58y0, B58y1, B58y2, B58y3, B58y4, B58y5, B58y6, B58y7, B58y8, B58y9, B58yz, B58z., B59.., B590., B591., B592., B592X, B593., B594., B59z., B59zX, B5y.., B5z.., B6..., B60.., B600., B6000, B6001, B6002, B6003, B6004, B6005, B6006, B6007, B6008, B600z, B601., B6010, B6011, B6012, B6013, B6014, B6015, B6016, B6017, B6018, B601z, B602., B6020, B6021, B6022, B6023, B6024, B6025, B6026, B6027, B6028, B602z, B60y., B60z., B61.., B610., B6100, B6101, B6102, B6103, B6104, B6105, B6106, B6107, B6108, B610z, B611., B6110, B6111, B6112, B6113, B6114, B6115, B6116, B6117, B6118, B611z, B612., B6120, B6121, B6122, B6123, B6124, B6125, B6126, B6127, B6128, B612z, B613., B6130, B6131, B6132, B6133, B6134, B6135, B6136, B6137, B6138, B613z, B614., B6140, B6141, B6142, B6143, B6144, B6145, B6146, B6147, B6148, B614z, B615., B6150, B6151, B6152, B6153, B6154, B6155, B6156, B6157, B6158, B615z, B616., B6160, B6161, B6162, B6163, B6164, B6165, B6166, B6167, B6168, B616z, B61z., B61z0, B61z1, B61z2, B61z3, B61z4, B61z5, B61z6, B61z7, B61z8, B61zz, B62.., B620., B6200, B6201, B6202, B6203, B6204, B6205, B6206, B6207, B6208, B620z, B621., B6210, B6211, B6212, B6213, B6214, B6215, B6216, B6217, B6218, B621z, B622., B6220, B6221, B6222, B6223, B6224, B6225, B6226, B6227, B6228, B622z, B623., B6230, B6231, B6232, B6233, B6234, B6235, B6236, B6237, B6238, B623z, B624., B6240, B6241, B6242, B6243, B6244, B6245, B6246, B6247, B6248, B624z, B625., B6250, B6251, B6252, B6253, B6254, B6255, B6256, B6257, B6258, B625z, B626., B6260, B6261, B6262, B6263, B6264, B6265, B6266, B6267, B6268, B626z, B627., B6270, B6271, B6272, B6273, B6274, B6275, B6276, B6277, B6278, B6279, B627A, B627B, B627C, B627D, B627E, B627W, B627X, B62x., B62x0, B62x1, B62x2, B62x3, B62x4, B62x5, B62x6, B62xX, B62y., B62y0, B62y1, B62y2, B62y3, B62y4, B62y5, B62y6, B62y7, B62y8, B62yz, B62z., B62z0, B62z1, B62z2, B62z3, B62z4, B62z5, B62z6, B62z7, B62z8, B62zz, B63.., B630., B6300, B6301, B6302, B6303, B631., B63y., B63z., B64.., B640., B641., B642., B64y., B64y0, B64y1, B64y2, B64yz, B64z., B65.., B650., B651., B6510, B6512, B651z, B652., B653., B6530, B6531, B653z, B65y., B65y0, B65y1, B65yz, B65z., B66.., B660., B661., B662., B66y., B66y0, B66yz, B66z., B67.., B670., B671., B672., B673., B674., B675., B67y., B67y0, B67yz, B67z., B68.., B680., B681., B682., B68y., B68z., B69.., B690., B691., B692., B6y.., B6y0., B6y1., B6z.., B6z0., B831., B8310, B8311, ByuFA, BB2K., BBG4., BBG5., BBGN., BBGP., BBm1., BBm4., BBM6., BBM7., BBM8., BBmA., BBmB., BBmC., BBmE., BBmF., BBmG., BBmK., BBmz., BBN0., BBN1., BBN2., BBN3., BBNz., BBrA6, BBrA7, BBrA8, BBs.., BBs0., BBS1., BBS2., BBs4., BBs5., BBsz., ByuD., ByuD4, ByuDA, ByuDB, BBR.., BBr0., BBr00, BBr01, BBr02, BBr03, BBr04, BBr0z, BBR1., BBr10, BBr1z, BBr2., BBr20, BBr21, BBr22, BBr23, BBr24, BBr25, BBr26, BBr27, BBr2z, BBr3., BBr30, BBr3z, BBR4., BBr40, BBr41, BBr42, BBr4z, BBR5., BBr50, BBr5z, BBR6., BBr60, BBr61, BBr62, BBr63, BBr64, BBr65, BBr66, BBr67, BBr68, BBr6z, BBR7., BBr70, BBr7z, BBr8., BBr80, BBr8z, BBr9., BBr90, BBr91, BBr92, BBr93, BBr94, BBr9z, BBrA., BBrA0, BBrA1, BBrA2, BBrA4, BBrA5, BBrAz, BBRz., ByuD5, ByuD6, ByuD7, ByuD8, ByuD9, BBg1., BBg10, BBg2., BBg3., BBg7., BBg8., BBg9., BBgA., BBgB., BBgC., BBgD., BBgE., BBgF., BBgG., BBgH., BBgJ., BBgK., BBgL., BBgM., BBgR., BBgS., BBgT., BBgV., BBgz., BBK.., BBk0., BBk1., BBK2., BBK3., BBk4., BBk5., BBk6., BBk7., BBk8., BBKz., BBM5., BBM9., BBmD., BBmH., BBQ.., BBQ0., BBQz., BBV0., BBV2., ByuD1, ByuD2, ByuD3, ByuDC, ByuDD, ByuDE, ByuDF, BBJ.., BBJ0., BBJ1., BBj10, BBj11, BBj2., BBJ3., BBj4., BBJ5., BBJ6., BBj60, BBj61, BBj62, BBJ7., BBJ8., BBJ9., BBJA., BBJz., ByuD0, BB..., BB0.., BB02., BB03., BB07., BB08., BB09., BB0A., BB0z., BB1.., BB12., BB13., BB14., BB16., BB17., BB18., BB19., BB1A., BB1B., BB1C., BB1D., BB1E., BB1F., BB1G., BB1H., BB1J., BB1K., BB1L., BB1M., BB1N., BB2.., BB20., BB22., BB24., BB26., BB2A., BB2B., BB2C., BB2D., BB2E., BB2F., BB2G., BB2H., BB2J., BB2M., BB2N., BB2z., BB3.., BB30., BB31., BB32., BB33., BB34., BB35., BB36., BB3z., BB4.., BB41., BB43., BB46., BB47., BB48., BB49., BB4A., BB50., BB500, BB52., BB520, BB53., BB54., BB55., BB56., BB57., BB58., BB5a1, BB5B., BB5B4, BB5C., BB5c0, BB5C0, BB5c2, BB5d1, BB5f., BB5f1, BB5f2, BB5f3, BB5f6, BB5f7, BB5h., BB5hz, BB5J., BB5j3, BB5j4, BB5K., BB5L1, BB5L2, BB5L3, BB5M1, BB5P., BB5Q., BB5R., BB5R0, BB5R1, BB5R2, BB5R3, BB5R4, BB5R5, BB5R6, BB5R8, BB5R9, BB5RA, BB5Rz, BB5S., BB5Sz, BB5T1, BB5U1, BB5U2, BB5V1, BB5V3, BB5V5, BB5V7, BB5W1, BB5X1, BB5y., BB5y0, BB5y1, BB5y3, BB5y5, BB5y6, BB6.., BB60z, BB611, BB621, BB63., BB64., BB65., BB66., BB67., BB68., BB6A1, BB7.., BB70., BB71., BB7z., BB8.., BB80., BB801, BB81., BB812, BB815, BB818, BB819, BB81A, BB81B, BB81E, BB81H, BB81J, BB81K, BB81L, BB81M, BB81z, BB821, BB83., BB84., BB85., BB850, BB851, BB85z, BB8z., BB9.., BB90., BB91., BB910, BB911, BB92., BB93., BB94., BB96., BB98., BB9B., BB9C., BB9D., BB9E0, BB9F., BB9G., BB9H., BB9J., BB9L., BB9M., BB9z., BBa.., BBA.., BBA1., BBA2., BBa3., BBa4., BBAz., BBB.., BBB0., BBB1., BBB2., BBB3., BBb4., BBB4., BBb5., BBB5., BBb7., BBB7., BBb8., BBb9., BBbA., BBbD., BBbE., BBbz., BBBz., BBc.., BBC.., BBc0., BBC0., BBc00, BBc1., BBC11, BBC12, BBC1z, BBc2., BBC2., BBc3., BBC3., BBC30, BBc4., BBC4., BBc5., BBC5., BBC6., BBC61, BBC6z, BBc8., BBc9., BBCA., BBCB., BBCC., BBcD., BBCD., BBCG., BBcz., BBCz., BBd.., BBd0., BBd1., BBd5., BBd7., BBd8., BBD8., BBd9., BBDA., BBdB., BBDB., BBDC., BBDD., BBDE., BBDF., BBdz., BBDz., BBe.., BBE.., BBe0., BBE0., BBe1., BBE2., BBe3., BBe4., BBe5., BBe6., BBe7., BBE7., BBe8., BBE8., BBe9., BBeA., BBEa., BBEF., BBez., BBf.., BBF.., BBf0., BBF0., BBf1., BBF1., BBf2., BBF2., BBF3., BBF4., BBF5., BBF6., BBfz., BBFz., BBg.., BBG.., BBG1., BBG2., BBG3., BBg6., BBG8., BBG9., BBGA., BBGB., BBGC., BBGD., BBGE., BBGF., BBGG., BBGH., BBGJ., BBGK., BBGL., BBGM., BBgN., BBgP., BBgQ., BBGz., BBh.., BBH.., BBh0., BBH0., BBh1., BBH1., BBh2., BBhz., BBHz., BBHZ., BBj.., BBj1., BBJ2., BBj3., BBJ4., BBj5., BBj6., BBj7., BBj8., BBJB., BBJB0, BBJB1, BBJB2, BBJB3, BBJBz, BBJC., BBJD., BBJE., BBJF., BBJH., BBjz., BBk.., BBK0., BBK00, BBK01, BBK02, BBK03, BBK04, BBK05, BBK06, BBK07, BBK0z, BBK1., BBK10, BBK11, BBK1z, BBk2., BBK20, BBK21, BBK2z, BBk3., BBK30, BBK34, BBK35, BBK38, BBK3z, BBkz., BBL.., BBl1., BBL1., BBL2., BBL3., BBL4., BBL5., BBL6., BBL70, BBL71, BBL72, BBL73, BBL9., BBLA., BBLB., BBLC., BBLC0, BBLC1, BBLCz, BBLD., BBLE., BBLG., BBLH., BBLJ., BBLz., BBm.., BBM.., BBm0., BBM01, BBM1., BBm2., BBM2., BBm3., BBM3., BBM4., BBm5., BBm6., BBm7., BBm8., BBm9., BBMA., BBMB., BBmJ., BBMz., BBn.., BBN.., BBn0., BBN4., BBN5., BBnz., BBp.., BBP.., BBP0., BBP1., BBp2., BBP8., BBP9., BBPX., BBpz., BBPz., BBq.., BBq0., BBQ2., BBQ3., BBQ4., BBQ5., BBQ6., BBQ7., BBQ71, BBQ72, BBQ73, BBQ74, BBQ75, BBQ7z, BBQA., BBQA0, BBQA2, BBQAz, BBQB., BBqz., BBrA3, BBS.., BBS0., BBs1., BBs2., BBs3., BBS3., BBSz., BBT0., BBT1., BBT2., BBT3., BBT4., BBT5., BBT7., BBT70, BBT71, BBT7z, BBT8., BBT9., BBTA., BBTB., BBTC., BBTD., BBTD0, BBTD1, BBTD2, BBTDz, BBTE., BBTF., BBTG., BBTH., BBTJ., BBTK., BBTL., BBTz., BBU.., BBU0., BBU1., BBU2., BBU3., BBU4., BBU5., BBU6., BBU7., BBUz., BBV.., BBV1., BBVz., BBW.., BBW0., BBW1., BBW2., BBW3., BBW4., BBW5., BBW6., BBW7., BBWA., BBWz., BBX.., BBX0., BBX2., BBX3., BBXz., BBY0., BBy1., BBY1., BBY2., BByz., BBYz., BBz.., BBZ.., BBZ1., BBZ2., BBZ3., BBZ4., BBZ6., BBZ7., BBZ8., BBZ9., BBZA., BBZC., BBZD., BBZE., BBZF., BBZJ., BBZK., BBZL., BBZM., BBZN., BBZP., BBZz., By..., Byu.., ByuC., ByuC0, ByuC1, ByuC2, ByuC3, ByuC4, ByuC5, ByuC6, ByuC7, ByuC8, ByuE., ByuE0, BBb.., BBb0., BBb1., BBb2., BBb3., BBbC., BBbR., BBbT., BBbU., BBbV., BBbW., BBbX., BBbZ., BBD2., ByuA0, ByuA1, ByuA2, ByuA3, BBcA., BBCC1, BBQ1., BBQ10, BBQ11, BBQ1z, Byu8., Byu80, Byu81, Byu82, BBQA1, Byu7., Byu70, Byu71, Byu72, Byu73, BB5j2, BB5j5, BBL0., BBR2., BBR3., BBcB., BBG7., BBK31, BBK32, BBK33, BBK36, BBK37, Byu5., Byu50, Byu51, Byu52, Byu53, Byu54, Byu55, Byu56, Byu57, Byu58, Byu59, Byu5B, BB601, BB612, BB691, BB6z., BBEV., BBl.., BBl0., BBlz., BBQ9., Byu4., Byu42, Byu43, Byu5A, BBE1., BBE10, BBE11, BBe2., BBE4., BBEA., BBEC., BBEE., BBEG., BBEG0, BBEH., BBEM., BBEP., BBEQ., BBER., BBES., BBET., BBEX., Byu40, Byu41, BBg5., BBn1., BBn2., BBn3., BBv1., BBv2., BBV3., BBV4., BBV5., BBV6., BBV8., BBV9., BBVA., BBW8., BBW9., BBX1., Byu3., Byu30, Byu31, Byu32, Byu33, BBp1., BBP3., BBP5., BBP7., Byu2., Byu21, Byu22, Byu23, Byu24, Byu25, BB5S2, BB5S4, Byu20, Byu1., Byu12, Byu13, BB5B1, BB5B2, BB5B3, BB5B5, BB5B6, BB5D., BB5D0, BB5D1, BB5D3, BB5D5, BB5D7, BB5D8, BB5Dz, Byu10, Byu11, BB5N., BB5N1, BB5C1, BB5Cz, BBZG., BBZH., Byu0. |  |
| Alcohol Problems^m^ | 13Y8., 1462, 1B1c., 66e.., 66e0., 8BA8., 8H35., 8H7p., 8HkG., 8HkJ., 9NN2., C1505, E01.., E010., E011., E0110, E0111, E011z, E012., E0120, E013., E014., E015., E01y., E01y0, E01yz, E01z., E23.., E230., E2300, E2301, E2302, E2303, E230z, E231., E2310, E2311, E2312, E2313, E231z, E23z., Eu101, Eu102, Eu103, Eu104, Eu105, Eu106, Eu107, Eu108, F11x0, F1440, F25B., F375., F3941, G555., J153., J610., J611., J612., J6120, J613., J6130, J617., J6170, J6710, Z1911, Z4B1. |  |
| Psychoactive Substance Misuse^m^ | 13c.., 13c0., 13c1., 13c2., 13c3., 13c4., 13c5., 13c6., 13c7., 13c8., 13c9., 13cA., 13cB., 13cC., 13cD., 13cE., 13cH., 13cK., 13cM., 13cN., 13cQ., 13cR., 13cS., 13cT., 1B1c., 1P30., 1P31., 1P6.., 1P60., 1P62., 1P63., 1P64., 1TE.., 1TF.., 68U.., 68U0., 8AA.., 8B23., 8B2N., 8B2P., 8B2Q., 8B2R., 8B2S., 8B2T., 8BA9., 8BAd., 8BE0., 8BE1., 8FB.., 8FB0., 9HC.., 9HC0., 9HC1., E02.., E020., E021., E0210, E0211, E021z, E022., E02y., E02y0, E02y1, E02y2, E02y3, E02y4, E02yz, E02z., E24.., E240., E2400, E2401, E2402, E2403, E240z, E241., E2410, E2411, E2412, E2413, E241z, E242., E2420, E2421, E2422, E2423, E242z, E243., E2430, E2431, E2432, E2433, E243z, E244., E2440, E2441, E2442, E2443, E244z, E245., E2450, E2451, E2452, E2453, E245z, E246., E2460, E2461, E2462, E2463, E246z, E247., E2470, E2471, E2472, E2473, E247z, E248., E2480, E2481, E2482, E2483, E248z, E249., E2490, E2491, E2492, E2493, E249z, E24A., E24z., E25.., E252., E2520, E2521, E2522, E2523, E252z, E253., E2530, E2531, E2532, E2533, E253z, E254., E2540, E2541, E2542, E2543, E254z, E255., E2550, E2551, E2552, E2553, E255z, E256., E2560, E2561, E2562, E2563, E256z, E257., E2570, E2571, E2572, E2573, E257z, E258., E2580, E2581, E2582, E2583, E258z, E259., E2590, E2591, E2592, E2593, E2594, E259z, E25y., E25y0, E25y1, E25y2, E25y3, E25yz, E25z., E24.., E240., E2400, E2401, E2402, E2403, E240z, E241., E2410, E2411, E2412, E2413, E241z, E242., E2420, E2421, E2422, E2423, E242z, E243., E2430, E2431, E2432, E2433, E243z, E244., E2440, E2441, E2442, E2443, E244z, E245., E2450, E2451, E2452, E2453, E245z, E246., E2460, E2461, E2462, E2463, E246z, E247., E2470, E2471, E2472, E2473, E247z, E248., E2480, E2481, E2482, E2483, E248z, E249., E2490, E2491, E2492, E2493, E249z, E24A., E24z., E25.., E252., E2520, E2521, E2522, E2523, E252z, E253., E2530, E2531, E2532, E2533, E253z, E254., E2540, E2541, E2542, E2543, E254z, E255., E2550, E2551, E2552, E2553, E255z, E256., E2560, E2561, E2562, E2563, E256z, E257., E2570, E2571, E2572, E2573, E257z, E258., E2580, E2581, E2582, E2583, E258z, E259., E2590, E2591, E2592, E2593, E2594, E259z, E25y., E25y0, E25y1, E25y2, E25y3, E25yz, E25z., Eu1.., Eu11., Eu110, Eu111, Eu112, Eu113, Eu114, Eu115, Eu116, Eu117, Eu11y, Eu11z, Eu12., Eu120, Eu121, Eu122, Eu123, Eu124, Eu125, Eu126, Eu127, Eu12y, Eu12z, Eu13., Eu130, Eu131, Eu132, Eu133, Eu134, Eu135, Eu136, Eu137, Eu13y, Eu13z, Eu14., Eu140, Eu141, Eu142, Eu143, Eu144, Eu145, Eu146, Eu147, Eu14y, Eu14z, Eu16., Eu160, Eu161, Eu162, Eu163, Eu164, Eu165, Eu166, Eu167, Eu16y, Eu16z, Eu18., Eu180, Eu181, Eu182, Eu183, Eu184, Eu185, Eu186, Eu187, Eu18y, Eu18z, Eu19., Eu190, Eu191, Eu192, Eu193, Eu194, Eu195, Eu196, Eu197, Eu19y, Eu19z, Eu1A., Eu1A0, Eu1A1, Eu1A2, Eu1A3, Eu1A4, Eu1A5, Eu1A6, Eu1A7, Eu1Ay, Eu1Az, ZV114 |  |
| Stroke or Transient Ischaemic Attack | 14AB., 14AK., 14A7., 1M4.., 662e., 662M., 662o., 7P242, 8HBJ., G61.., G610., G611., G612., G613., G614., G615., G616., G617., G618., G61X., G61X0, G61X1, G61z., G62.., G620., G621., G622., G623., G62z., G63y0, G63y1, G64.., G6400, G6410, G64z., G64z0, G64z2, G64z3, G64z4, G65.., G650., G651., G6510, G652., G653., G654., G656., G65y., G65z., G65z0, G65z1, G65zz, G66.., G663., G664., G667., G668., G669., G6760, G6W.., G6X.., Gyu62, Gyu63, Gyu64, Gyu65, Gyu66, Gyu6F, Gyu6G, ZV12D, Fyu55 |  |
| Atrial Fibrillation | 14AN., 3272, 662S., 6A9.., G573., G5730, G5732, G5733, G5734, G5735, G573z |  |
| Peripheral Vascular Disease | 14NB., G73.., G730., G7300, G7301, G730z, G731., G7310, G7311, G731z, G732., G7320, G7321, G7322, G7323, G7324, G733., G73y., G73y0, G73y1, G73y2, G73y4, G73y5, G73y6, G73y7, G73y8, G73yz, G73z., G73z0, G73zz, Gyu74, P76.., G830., G831., G832., G833., G835., G836., G837., G8y1., G8y3., G8yy0, G702., G702z |  |
| Heart Failure | 14A6., 14AM., 1O1.., 662p., 662T., 662W., 8B29., 8H2S., 9N0k., G1yz1, G58.., G580., G5800, G5801, G5802, G5803, G5804, G581., G5810, G582., G583., G58z., G5y4z, L09y2, Q48y1 |  |
| Prostate Disorders | 8L51., A1650, A9812, A9832, AD103, B46.., B58y5, B7C2., B834., B8340, B915., K20.., K200., K201., K202., K20z., K21.., K210., K211., K212., K213., K214., K2140, K2141, K2142, K2143, K2144, K2145, K2146, K214z, K21y., K21z., K22.., K220., K221., K2210, K2211, K221z, K222., K22y., K22y0, K22y1, K22y2, K22y3, K22yz, K22z., Kyu60, Kyu61, Kyu68, PCy01, PCyx., Pyu69 |  |
| Glaucoma | 66T1., 7275, F4421, F45.., F450., F4501, F4502, F4503, F450z, F451., F4510, F4511, F4512, F4513, F4514, F4515, F451z, F452., F4520, F4521, F4522, F4523, F4524, F452z, F453., F4530, F4531, F453z, F454., F4540, F4541, F4542, F4543, F4544, F454z, F455., F4550, F4551, F455z, F456., F4560, F4561, F4562, F4563, F4564, F4565, F4566, F456z, F45y., F45y0, F45y1, F45y2, F45yz, F45z., F4631, F4H14, FyuG., FyuG0, FyuG1, FyuG2, P3200, Q20y7 |  |
| Epilepsy | 1473, 1B1W., 1O30., 667.., 6671, 6672, 6673, 6674, 6675, 6676, 6677, 6678, 6679, 667D., 667E., 667G., 667H., 667J., 667K., 667L., 667M., 667N., 667Q., 667R., 667S., 667T., 667V., 667W., 667X., 667Z., 8B66., 8BIF., Eu803, F1321, F25.., F250., F2500, F2501, F2502, F2503, F2504, F2505, F250y, F250z, F251., F2510, F2511, F2512, F2513, F2514, F2515, F2516, F251y, F251z, F252., F253., F254., F2540, F2541, F2542, F2543, F2544, F2545, F254z, F255., F2550, F2551, F2552, F2553, F2554, F2555, F2556, F255y, F255z, F256., F2560, F2561, F256z, F257., F258., F259., F25A., F25B., F25C., F25D., F25E., F25F., F25X., F25y., F25y0, F25y1, F25y2, F25y3, F25y4, F25y5, F25yz, F25z., Fyu50, Fyu51, Fyu52, Fyu59, SC200 | AND Four or more prescriptions for antiepilpetics issued per year. Medication read codes:  dnc.. , dnc1. , do... , do1.. , do11. , do12. , do14. , do15. , do16. , do18. , do19. , do1A. , do1B. , do1t. , do1u. , do1v. , do1w. , do1x. , do1y. , dn2.. , dn21. , dn2z. , dnx.. , dnx2. , dnx4. , dnx6. , dnx8. , dnxA. , dnxC. , dnxE. , dn3.. , dn31. , dn32. , dn33. , dn34. , dn35. , dn36. , dn37. , dn38. , dn39. , dn3a. , dn3A. , dn3b. , dn3B. , dn3c. , dn3C. , dn3d. , dn3D. , dn3e. , dn3E. , dn3f. , dn3F. , dn3G. , dn3H. , dn3I. , dn3J. , dn3K. , dn3v. , dn3w. , dn3x. , dn3y. , dn3z. , dn4.. , dn41. , dn42. , dn4w. , dn4x. , dn4y. , dn4z. , do2.. , do21. , do2z. , dnu.. , dnu1. , dnu2. , dn5.. , dn53. , dn54. , dn55. , dn56. , dn5x. , dn5y. , dn5z. , dni.. , dni1. , dni2. , dnj.. , dnj1. , dnj2. , dnj3. , dnj4. , dnj5. , dnj6. , dnj7. , dnj8. , dnj9. , dnjA. , dnjx. , dnjy. , dnjz. , dnt.. , dnt1. , dnt2. , dnt3. , dnt4. , dnt5. , dnt6. , dnt7. , dnt8. , dnt9. , dntA. , dntB. , dntC. , dntD. , dntE. , dnf.. , dnf1. , dnf2. , dnf3. , dnf4. , dnf5. , dnf6. , dnf7. , dnf8. , dnf9. , dnfA. , dnfB. , dnfC. , dnfD. , dnfE. , dnfF. , dnfG. , dnfH. , dnfJ. , dnfz. , dno.. , dno1. , dno2. , dno3. , dno4. , dno5. , dno6. , dno7. , dno8. , dno9. , dnor. , dnos. , dnot. , dnou. , dnov. , dnow. , dnox. , dnoy. , dnoz. , dn6.. , dn61. , dn62. , dn63. , dn6x. , dn6y. , dn6z. , dnm.. , dnm1. , dnm2. , dnm3. , dnm4. , dnmw. , dnmx. , dnmy. , dnmz. , dnw.. , dnw1. , dnw2. , dnw3. , dnw4. , dnw5. , dnw6. , dnwu. , dnwv. , dnww. , dnwx. , dnwy. , dnwz. , dn7.. , dn71. , dn72. , dn73. , dn74. , dn75. , dn76. , dn77. , dn78. , dn79. , dn7a. , dn7b. , dn7c. , dn7d. , bc6.. , bc61. , bc62. , dn8.. , dn81. , dn82. , dn83. , dn8y. , dn8z. , dn9.. , dn91. , dn92. , dn93. , dn94. , dn95. , dn96. , dn97. , dn98. , dn9w. , dn9x. , dn9y. , dn9z. , do6.. , do61. , do6z. , dnp.. , dnp1. , dnp2. , dnp3. , dnp4. , dnp5. , dnp6. , dnp7. , dnp8. , dnp9. , dnpr. , dnps. , dnpt. , dnpu. , dnpv. , dnpw. , dnpx. , dnpy. , dnpz. , dna.. , dna1. , dna2. , dna3. , dnax. , dnay. , dnaz. , dnv.. , dnv1. , dnv2. , dnv3. , dnv4. , dnv5. , dnv6. , dnv7. , dnv8. , dnv9. , dnvA. , dnvB. , dnvC. , dnr.. , dnr1. , dnr2. , dnr3. , dnr4. , dnrw. , dnrx. , dnry. , dnrz. , dns.. , dns1. , dns2. , dns3. , dns4. , dnsw. , dnsx. , dnsy. , dnsz. , dnl.. , dnl1. , dnl2. , dnl3. , dnl4. , dnl5. , dnl6. , dnk.. , dnk1. , dnk2. , dnk3. , dnk4. , dnk5. , dnk6. , dnk7. , dnk8. , dnk9. , dnkA. , dnkB. , dnkC. , dnkD. , dnkE. , dnb.. , dnb1. , dnb2. , dnb3. , dnb4. , dnb5. , dnb6. , dnb7. , dnb8. , dnb9. , dnba. , dnbA. , dnbb. , dnbB. , dnbc. , dnbC. , dnbd. , dnbD. , dnbe. , dnbE. , dnbF. , dnbG. , dnbH. , dnbI. , dnbJ. , dnbK. , dnbL. , dnbM. , dnbn. , dnbN. , dnbo. , dnbO. , dnbp. , dnbP. , dnbq. , dnbQ. , dnbr. , dnbR. , dnbs. , dnbS. , dnbt. , dnbT. , dnbu. , dnbU. , dnbv. , dnbw. , dnbx. , dnby. , dnbz. , dnh.. , dnh1. , dnh2. , dnh3. , dnh4. , dnh5. , dnh6. , dnh7. , dnh8. , dnhy. , dnhz. , dne.. , dne1. , dne2. , dne3. , dne4. , dnq.. , dnq1. , dnq2. , dnq3. , dnq4. , dnq5. , dnq6. |
| Dementia^m^ | 66h.., 6AB.., E000., E001., E0010, E0011, E0012, E0013, E001z, E002., E0020, E0021, E002z, E003., E004., E0040, E0041, E0042, E0043, E004z, E012., E02y1, E041., Eu00., Eu000, Eu001, Eu002, Eu00z, Eu01., Eu010, Eu011, Eu012, Eu013, Eu01y, Eu01z, Eu02., Eu020, Eu021, Eu022, Eu023, Eu024, Eu025, Eu02y, Eu02z, Eu041, F110., F1100, F1101, F111., F112., F116., Fyu30 |  |
| Schizophrenia and Bipolar Affective Disorder^m^ | 1464, E10.., E100., E1000, E1001, E1002, E1003, E1004, E1005, E100z, E101., E1010, E1011, E1012, E1013, E1014, E1015, E101z, E102., E1020, E1021, E1022, E1023, E1024, E1025, E102z, E103., E1030, E1031, E1032, E1033, E1034, E1035, E103z, E104., E105., E1050, E1051, E1052, E1053, E1054, E1055, E105z, E106., E107., E1070, E1071, E1072, E1073, E1074, E1075, E107z, E10y., E10y0, E10y1, E10yz, E10z., E11.., E110., E1100, E1101, E1102, E1103, E1104, E1105, E1106, E110z, E111., E1110, E1111, E1112, E1113, E1114, E1115, E1116, E111z, Eu2.., Eu20., Eu200, Eu201, Eu202, Eu203, Eu204, Eu205, Eu206, Eu20y, Eu20z, Eu21., Eu22., Eu220, Eu221, Eu222, Eu223, Eu22y, Eu22z, Eu23., Eu230, Eu231, Eu232, Eu233, Eu23y, Eu23z, Eu24., Eu25., Eu250, Eu251, Eu252, Eu25y, Eu25z, Eu26., Eu2y., Eu2z., ZV110, E114., E1140, E1141, E1142, E1143, E1144, E1145, E1146, E114z, E115., E1150, E1151, E1152, E1153, E1154, E1155, E1156, E115z, E116., E1160, E1161, E1162, E1163, E1164, E1165, E1166, E116z, E117., E1170, E1171, E1172, E1173, E1174, E1175, E1176, E117z, E11y., E11y0, E11y1, E11y2, E11y3, E11yz, Eu31., Eu310, Eu311, Eu312, Eu313, Eu314, Eu315, Eu316, Eu317, Eu31y, Eu31z | OR Four or more prescriptions for antipsychotics issued per year. Medication read codes:  d6... , d61.. , d611. , d612. , d613. , d614. , d615. , d616. , d617. , d618. , d619. , d61s. , d61v. , d61w. , d61x. , d61y. , d61z. , d62.. , d621. , d622. , d623. , d624. , d625. , d62w. , d62x. , d62y. , d62z. |
| Psoriasis and Eczema | 14F2., M160., M1600, M1601, M160z, M161., M1610, M1611, M1612, M1613, M1614, M1615, M1616, M1617, M1618, M1619, M161A, M161B, M161C, M161D, M161E, M161F, M161G, M161H, M161z, M16y., M16y0, M16z., Myu30, N0452, Nyu13, 14F1., 26C4., F4D30, F5024, M102., M111., M112., M113., M114., M119., M11A., M12z1, M12z2, M12z3, M12z4, M1y2., Myu2., Myu22 | AND Four or more prescriptions for creams issued per year. Medication read codes:  m4... , m4b5. , m51.. , m513. , m514. , m518. , m519. , m51A. , m51c. , m51C. , m51d. , m51F. , m51G. , m51h. , m51H. , m51I. , m51l. , m51L. , m51m. , m51R. , m51T. , m51u. , m51v. , m5D.. , m5D1. , m5G.. , m5G1. , m5G2. , m5G3. , m5G4. , m5G5. , m5G6. , m5G7. , m5G8. , mb51. , m5A.. , m5A1. , m5A2. , m5A3. , m5A4. , mh1k. , mh1l. , m46.. , m461. , m462. , m463. , m464. , m46y. , m46z. , m47.. , m479. , m47a. , m47y. , m47z. , m48.. , m482. , m483. , m485. , m486. , m487. , m489. , m48a. , m48A. , m48b. , m48B. , m48c. , m48C. , m48d. , m48D. , m48e. , m48E. , m48f. , m48g. , m48h. , m48i. , m48j. , m48k. , m48l. , m48m. , m48n. , m48o. , m48p. , m48q. , m48r. , m48s. , m48t. , m48z. , m49.. , m496. , m497. , m499. , m49a. , m49c. , m49d. , m49e. , m49f. , m49r. , m49s. , m49t. , m49u. , m49v. , m49w. , m49x. , m49y. , m49z. , me46. , me4D. , me4x. , m492. , m494. , m4o.. , m4o1. , m4o2. , m4o3. , m4o4. , m4o5. , m4o6. , m4o7. , m4o8. , m59.. , m591. , m592. , m593. , m594. , m595. , m596. , m597. , m598. , m599. , m59A. , m59B. , m59C. , m59D. , m59E. , m59F. , m59G. , m59H. , m59I. , m59J. , m59K. , m59L. , m59M. , ip29. , ip2A. , ip2B. , m4a.. , m4a1. , m4a2. , m4a3. , m4a4. , m4a5. , m4a6. , m4a9. , m4aa. , m4ab. , m4ac. , m4ad. , m4ae. , m4af. , m4ag. , m4ah. , m4ai. , m4aj. , m4ak. , m4al. , m4aw. , m4ax. , m4ay. , m4az. , m4a7. , m4a8. , m4b.. , m4b1. , m4b2. , m4b3. , m4b4. , m4b7. , m4by. , m4bz. , m4c.. , m4c1. , m4c2. , m4c3. , m4cy. , m4cz. , m4d.. , m4d1. , m4d2. , m4d4. , m4d5. , m4dy. , m4dz. , m4e.. , m4e1. , m4e2. , m4e3. , m4e4. , m4e5. , m4e6. , m4e7. , m4e8. , m4eu. , m4ev. , m4ew. , m4ex. , m4ey. , m4ez. , m53.. , m531. , m532. , m533. , m534. , m535. , m536. , m537. , m53a. , m53b. , m53c. , m53d. , m53n. , m53o. , m53p. , m53q. , m53r. , m53s. , m53t. , m54.. , m5z.. , m5z1. , m5z2. , m5z3. , m5z4. , m5z5. , m5z6. , m5z7. , m5z8. , m5z9. , m5zm. , m5zn. , m5zo. , m5zp. , m5zq. , m5zr. , m5zs. , m5zt. , m5zu. , m5zv. , m5zw. , m5zx. , m5zy. , m5zz. , m53A. , m53B. , m53C. , m53e. , m53f. , m53F. , m53g. , m53h. , m53i. , m53k. , m53l. , m53m. , m55.. , m551. , m552. , m55y. , m55z. , m4f.. , m4f1. , m4f2. , m4f3. , m4f4. , m4fy. , m4fz. , m4j.. , m4j1. , m4j2. , m4j7. , m4j8. , m4ju. , m4jv. , m4jw. , m4jx. , m4jy. , m4jz. , m4j5. , m4j6. , m4g.. , m4g1. , m4g2. , m4g3. , m4g4. , m4g5. , m4g6. , m4g7. , m4g8. , m4g9. , m4gi. , m4gj. , m4gs. , m4gt. , m4gu. , m4gv. , m4gw. , m4gz. , m4ge. , m4gf. , m4gg. , m4gh. , m4go. , m4gp. , m4gq. , m4gr. , m4gc. , m4gd. , m4h.. , m4h1. , m4h2. , m4h3. , m4h4. , m4h5. , m4hx. , m4hy. , m4hz. , m4i.. , m4i1. , m4i3. , m4i6. , m4i8. , m4i9. , m4ia. , m4ib. , m4ic. , m4id. , m4ie. , m4if. , m4ig. , m4r.. , m4r1. , m4r2. , m4r3. , m4r4. , m4r5. , m4r6. , m4r7. , m4r8. , m4r9. , m4rA. , m4k.. , m4k1. , m4kz. , m41.. , m411. , m412. , m413. , m414. , m415. , m416. , m417. , m418. , m419. , m41a. , m41A. , m41b. , m41B. , m41c. , m41C. , m41d. , m41D. , m41e. , m41f. , m41k. , m41l. , m41m. , m41n. , m41o. , m41p. , m41q. , m41t. , m41u. , m41v. , m41w. , m42.. , m421. , m422. , m423. , m424. , m429. , m42a. , m42A. , m42b. , m42B. , m42c. , m42C. , m42d. , m42D. , m42e. , m44.. , m441. , m442. , m443. , m444. , m446. , m447. , m448. , m44c. , m44d. , m44e. , m44f. , m4p1. , m44a. , m44b. , m45.. , m451. , m452. , m453. , m454. , m455. , m457. , m458. , m459. , m45a. , m45A. , m45b. , m45B. , m45c. , m45C. , m45d. , m45e. , m45g. , m45k. , m45l. , m45m. , m45n. , m45o. , m45p. , m45q. , m45r. , m45s. , m45t. , m45T. , m45u. , m45v. , m45V. , m45w. , m45W. , m45x. , m45X. , m45y. , m45Y. , m45Z. , m44z. , m4l.. , m4l1. , m4l2. , m4l3. , m4l4. , m4l5. , m4l6. , m4l7. , m4la. , m4lb. , m4lt. , m4lw. , m4lx. , m4ly. , m4lz. , m4l8. , m4l9. , m4m.. , m4m1. , m4my. , m4mz. , m4q.. , m4q1. , m4q2. , m4q3. , m4q4. , m4q5. , m4q6. , m4q7. , m4q8. , m4q9. , m4qA. , m4qB. , m5B.. , m5B1. , m5B2. , m5B3. , m5B4. , m5B5. , m5B6. , m5C.. , m5C1. , m5C2. , m5C3. , m5C4. , m5C5. , m5C6. , m4n.. , m4n1. , m4n2. , m4n3. , m4n4. , m4n5. , m4n6. , m4ny. , m4nz. , m4n7. , m4n9. , m4na. , m4nb. , m4ne. , m4ng. , m4nv. |
| Inflammatory Bowel Disease | 14C4., J08z9, J40.., J400., J4000, J4001, J4002, J4003, J4004, J4005, J400z, J401., J4010, J4011, J4012, J401z, J402., J40z., J41.., J410., J4100, J4101, J4102, J4103, J4104, J410z, J411., J412., J41y., J4212, J4213, J42z0, J4302, J4303, J4312, J4313, J4322, J4323, J4332, J4333, J436., J4360, J4361, J437., J4z3., J4z5., J4z6., Jyu4., Jyu40, Jyu41, N0310, N0454, J08z9, J40.., J4002, J4003, J4004, J4005, J400z, J4012, J401z, Jyu40, N0311, N0453 |  |
| Hearing Loss | 1493, 1C12., 1C13., 1C131, 1C132, 1C133, 1C16., 1C18., 1C19., 1C1Z., 2BL2., 2BL3., 2BL4., 2BL5., 2BM2., 2BM3., 2BM4., 2DG.., 2DH0., 31343, 31344, 31345, 31346, F5801, F5812, F582., F59.., F590., F5900, F5901, F5902, F5903, F5904, F5905, F5906, F590y, F590z, F591., F5910, F5911, F5912, F5913, F5914, F5915, F5916, F5917, F5918, F591y, F591z, F592., F5920, F5921, F593., F594., F595., F596., F597., F598., F599., F59A., F59y., F59z., F5A.., FyuU0, FyuU1, P40.., P400., P402., P402z, P40z., P40zz, ZE63., ZE7.., ZE812, ZE813, ZE822, ZE823, ZE832, ZE833, ZE842, ZE843, ZE86., ZE87., ZV412 |  |
| Chronic Sinusitis | H13.., H130., H131., H132., H133., H134., H135., H13y., H13y0, H13y1, H13yz, H13z., Hyu22, J0835 |  |
| Anorexia Nervosa and Bulimia^m^ | 1467, E271., E2751, Eu500, Eu501, Eu502, Eu503 |  |
| Bronchiectasis | A115., H34.., H340., H341., H34z., P861. |  |
| Parkinson’s Disease | 147F., 297ª., 2987, 2994, A94y1, Eu023, F11x9, F12.., F120., F121., F123., F124., F12W., F12X., F12z., F1303, Fyu20, Fyu21, Fyu22, Fyu29, Fyu2B |  |
| Multiple Sclerosis | 666ª., 666B., 8CS1., F20.., F200., F201., F202., F203., F204., F205., F206., F207., F208., F20z. |  |
| Viral Hepatitis | 141E., 65Q7., A702., A7020, A703., A7030, A7040, A7050, A7051, A7054, A707., A7070, A7071, A7072, A707X, A70z0, AE23., AyuB1, AyuB2, AyuJ9, Q409., Q4091, Q409y, Q409z, ZV026, ZV02B, ZV02C |  |
| Chronic Liver Disease | G8522, J601., J6010, J6011, J6012, J601z, J60z., J61.., J614., J6140, J6141, J6142, J6143, J6144, J614y, J614z, J615., J6150, J6151, J6152, J6153, J6154, J6155, J6156, J6157, J6158, J6159, J615A, J615B, J615C, J615D, J615E, J615F, J615G, J615H, J615y, J615z, J616., J6160, J6161, J6162, J616z, J617., J6170, J61y., J61y0, J61y1, J61y2, J61y3, J61y4, J61y5, J61y6, J61y7, J61y8, J61yz, J61z., J623., J624., J62y., J62z., J630., Jyu71, PB62., PB620 |  |
| Diverticular Disease | 77180, J23z3, J5126, J5111, J51z., J5115, J512y, J5127, J511., J5113, J511z, J5116, J5125, J5102, J510z, J5108, J5117, J5124, J510., J5112, J51.., J513., J5103, J512., J5128, J5100, J5123, J5104, J5122, J512z, J5120, J511y, J5107, J5114, J5109, J5101, J5121, J5105, J510y, J5106, J5110 |  |
| Osteoporosis | 585O., 584E., 58E8., 58EA., 58EE., 58EG., 58EK., 58ES., 58EM., 58EV., 7230ª, 7230B, 7230D, 7230PM, 7230PT, N330., N330000, N330100, N330200, N330400, N330500, N330600, N330700, N330800, N330900, N330A00, N330B00, N330C00, N330C00, N330D00, N330z00, N331200, N331300, N331400, N331500, N331600, N331800, N331900, N331A00, N331B00, N331M00, N331N00, NyuB100, NyuB200, NyuB800 |  |
| Pernicious Anaemia | D010., F381500 |  |
| Endometriosis | 7E0D800, BL1., K50.., K500., K500000, K500100, K500111, K500200, K500z00, K501., K502., K503., K503000, K503100, K503200, K503300, K503z00, K504., K504., K504000, K504100, K504z00, K505., K505000, K505100, K505200, K505z00, K506.00, K50y.00, K50y000, K50y100, K50y200, K50y300, K50yz00, K50., Kyu9000 |  |
| Chronic Fatigue Syndrome | 8Q1.., F286., F286000, F286100, F286200 |  |
| Polycystic Ovarian Syndrome | C164., C165. |  |
| Meniere’s Disease | 1491, F560000, F560100, F560200, F560300, F560400, F560., F560z00 |  |
| Constipation |  | Four or more prescriptions for laxatives issued per year. Medication read codes:  ab... , ab1.. , ab13. , ab14. , ac... , ac6.. , ac61. , ad... , ae... , ae4.. , ae41. , ae42. , ae43. , ae44. , ae45. , ae46. , ae4a. , ae4h. , af... , af1.. , af1f. , af1k. , af1o. , af1q. , af1v. , ag... , ag1.. , l41b. , ac1.. , ac1.. , ac11. , ac11. , ac12. , ac13. , ac14. , ac15. , ac16. , af11. , af11. , af12. , af12. , af1a. , af1b. , af1c. , ac2.. , ac21. , ac3.. , ac31. , af1C. , ac4.. , ac42. , ac43. , ac44. , ac45. , ac46. , ac47. , ac48. , ac49. , ac4A. , ac4B. , ac4C. , ac4D. , ac4E. , ac4F. , ac4G. , ac5.. , ac51. , ac52. , ac53. , ac54. , ac55. , ac56. , ac57. , ac58. , ac59. , ac5A. , ac5B. , af1e. , af1p. , af1t. , af13. , af14. , af15. , ab2.. , ab21. , ab22. , ab23. , ab24. , ab25. , ab26. , ab28. , ab29. , ab2A. , ab2B. , ab2C. , ab2D. , ab2E. , ab2F. , ab2G. , ab2H. , ab2J. , ab2K. , ab2L. , ab2M. , ab2n. , ab2N. , ab2o. , ab2O. , ab2p. , ab2P. , ab2q. , ab2Q. , ab2r. , ab2R. , ab2t. , ab2u. , ab2v. , ab2w. , ab2x. , ab2y. , ae6.. , ae61. , ae6z. , ae1.. , ae11. , ae12. , ae13. , ae14. , ae15. , ae16. , ae17. , ae18. , ae19. , ae1A. , ae1B. , af1l. , af1m. , a4i.. , a4i1. , a4i2. , ad1.. , ad11. , ad12. , ad13. , ae21. , ag11. , ag15. , ag19. , ag1A. , ag1H. , ae47. , ae48. , ae49. , ae4g. , ae4b. , ae4c. , ae4d. , ae4e. , ae4f. , ae4i. , ae4j. , ae5.. , ae51. , ae3.. , ae31. , ae32. , ae33. , ag17. , ab3.. , ab32. , ab33. , ab35. , ab36. , ab37. , ax1.. , iz1D. , ax2.. , ax23. , ax24. , af1d. , af1s. , af1n. , ag12. , ag16. , ag1C. , ag1E. , ac7.. , ac71. , ac74. , ac75. , ac76. , ac77. , ac78. , ac79. , ac7A. , ac7v. , ac7w. , ac7x. , ac7y. , ac7z. , ae7.. , ae71. , af16. , af1A. , af1B. , af1g. , af1h. , af1w. , af1x. , af1y. , ac8.. , ac81. , ac82. , ac84. , ac85. , ac8w. , ac8x. , ac8y. , ac83. , ac86. , ac8z. , ab4.. , ab41. , ab42. , ab4y. , ab4z. , ab43. , ab44. , ab45. , ab46. , ab4x. |
| Dyspepsia |  | Four or more prescriptions for dyspepsia medication issued per year. Medication read codes:  a22w. , a22x. , a22y. , a22z. , a23K. , a23L. , a23M. , a23P. , a23Q. , a6... , a67.. , a671. , a6g.. , a6g1. , a6g2. , a6g3. , a6g4. , a22u. , a22v. , a23A. , a23b. , a23B. , a23c. , a23C. , a23D. , a23e. , a23E. , a23f. , a23F. , a23G. , a23H. , a23i. , a23I. , a23j. , a23J. , a23k. , a23l. , a23m. , a23n. , a23N. , a23o. , a23O. , a23p. , a23q. , a23s. , a23t. , a23v. , a23w. , a23x. , a23y. , a23z. , a24z. , a642. , a643. , a64y. , a64z. , a66.. , a661. , a662. , a66z. , a663. , a664. , a61.. , a611. , a612. , a613. , a614. , a615. , a616. , a617. , a618. , a619. , a61A. , a61B. , a61C. , a61d. , a61D. , a61e. , a61E. , a61f. , a61F. , a61g. , a61G. , a61H. , a61I. , a61J. , a61K. , a61L. , a61M. , a61N. , a61O. , a61P. , a61Q. , a61R. , a61S. , a61u. , a61v. , a61w. , a61x. , a61y. , a61z. , a61a. , a61b. , a61s. , a61t. , a61T. , a61U. , a6h.. , a6h1. , a6h2. , a6h3. , a6h4. , a6h5. , a6h6. , a6hu. , a6hv. , a6hw. , a6hx. , a6hy. , a6hz. , a24x. , a68.. , a681. , a682. , a683. , a684. , a6c.. , a6c1. , a6c2. , a6c3. , a6c4. , a6c5. , a6c6. , a6c7. , a6c8. , a6c9. , a6cA. , a6a.. , a6a1. , a6a2. , a69.. , a691. , a692. , a693. , a694. , a695. , a696. , a697. , a698. , a6b.. , a6b1. , a6b2. , a6b3. , a6b4. , a6b5. , a6b6. , a6b7. , a6b8. , a6b9. , a6bA. , a6bB. , a6bC. , a6bD. , a6bE. , a6bF. , a6bG. , a6bH. , a6bI. , a6bJ. , a6bK. , a6bL. , a6bM. , a6bN. , a6bO. , a6bP. , a6bQ. , a6bR. , a6bS. , a6bu. , a6bv. , a6bw. , a6bx. , a6by. , a6bz. , a6e.. , a6e1. , a6e2. , a6e3. , a6e4. , a6e5. , a6e6. , a6e7. , a63.. , a631. , a63z. , a6f.. , a6f1. , a6f2. , a6f3. , a6f4. , a62.. , a621. , a622. , a623. , a624. , a625. , a626. , a627. , a628. , a629. , a62A. , a62B. , a62C. , a62D. , a62E. , a62F. , a62G. , a62H. , a62I. , a62J. , a62K. , a62L. , a62M. , a62N. , a62O. , a62P. , a62Q. , a62u. , a62v. , a62w. , a62x. , a62y. , a62z. , a6d.. , a6d1. , a6d2. , a65.. , a651. , a652. , a65y. , a65z. EXCEPT if also administered non-steroidal anti-inflammatory drugs and/or antiplatelets. Medication read codes:, bu... , bu9.. , bu91. , bu92. , bu93. , bu94. , j28H. , j28z. , bu3.. , bu31. , bu32. , bu2.. , bu21. , bu22. , bu23. , bu24. , bu25. , bu26. , bu27. , bu28. , bu29. , bu2a. , bu2A. , bu2b. , bu2B. , bu2c. , bu2C. , bu2d. , bu2D. , bu2E. , bu2F. , bu2G. , bu2H. , bu2I. , bu2J. , bu2K. , bu9.. , bu91. , bu93. , bu5.. , bu51. , bu52. , bu53. , bu54. , bu55. , bu4.. , bu41. , bu42. , j2t.. , j2t1. , j2t2. , j2ty. , j2tz. , j2q.. , j2q1. , j2qz. , bu1.. , bu11. , bu12. , bu13. , bu14. , bu15. , bu16. , bu17. , bu18. , bu19. , bu1A. , bu1B. , bu1C. , bu1D. , bu1E. , bu1z. , br5.. , br51. , br52. , br53. , br54. , bu7.. , bu71. , bu72. , bu7y. , bu7z. , j25.. , j252. , j253. , j254. , j255. , j256. , j257. , j258. , j259. , j25A. , j25B. , j25y. , j25z. , di5.. , di51. , di5z. , j26.. , j261. , j262. , j263. , j264. , j27.. , j271. , j272. , j273. , j274. , j275. , j27x. , j27y. , j27z. , lf32. , di6.. , diaW. , j28.. , j281. , j282. , j283. , j284. , j285. , j286. , j287. , j288. , j289. , j28a. , j28A. , j28b. , j28B. , j28c. , j28C. , j28d. , j28D. , j28e. , j28E. , j28f. , j28F. , j28g. , j28G. , j28h. , j28i. , j28j. , j28J. , j28k. , j28K. , j28l. , j28m. , j28M. , j28n. , j28N. , j28o. , j28p. , j28P. , j28q. , j28r. , j28R. , j28s. , j28S. , j28t. , j28T. , j28u. , j28v. , j28w. , j28W. , j28x. , j28X. , j28y. , j28Y. , j28Z. , j2p.. , j2p1. , j2p2. , j2p3. , j2p4. , j2p5. , j2p6. , j2p7. , j2p8. , j2p9. , j2pa. , j2pA. , j2pb. , j2pB. , j2pc. , j2pC. , j2pD. , j2pE. , j2pF. , j2pG. , j2pH. , j2pI. , j2pJ. , j2pK. , j2pL. , j2pN. , j2pO. , j2pP. , j2pQ. , j2pR. , j2pS. , j2pU. , j2pV. , j2pW. , j2pX. , j2pY. , j2pZ. , dicw. , dicZ. , j28I. , j28O. , j28U. , j28V. , j2pM. , j2pT. , bn7.. , bn71. , bn72. , bn73. , bn74. , bn75. , j2a.. , j2a1. , j2a2. , j2a3. , j2a4. , j2a5. , j2a6. , j2a7. , j2a8. , j2a9. , j2aa. , j2aA. , j2ab. , j2aB. , j2aC. , j2aD. , j2aE. , j2aF. , j2aG. , j2aH. , j2aI. , j2aJ. , j2aK. , j2aL. , j2aM. , j2aO. , j2aP. , j2aQ. , j2aR. , j2aS. , j2aw. , j2ax. , j2ay. , j2az. , j2ac. , j2ad. , j2au. , j2av. , di8.. , di81. , di8z. , j2c.. , j2c1. , j2c2. , j2c3. , j2c4. , j2c5. , j2c6. , j2c7. , j2c8. , j2c9. , j2ca. , j2cA. , j2cb. , j2cB. , j2cc. , j2cC. , j2cd. , j2cD. , j2ce. , j2cE. , j2cf. , j2cF. , j2cg. , j2cG. , j2ch. , j2cH. , j2ci. , j2cI. , j2cj. , j2cJ. , j2ck. , j2cK. , j2cl. , j2cL. , j2cm. , j2cM. , j2cn. , j2cN. , j2co. , j2cO. , j2cp. , j2cq. , j2cr. , j2cs. , j2ct. , j2cu. , j2cv. , j2cw. , j2cx. , j2cy. , j2cz. , j2cP. , j2cZ. , buA.. , buA2. , buA4. , j2g.. , j2g1. , j2g2. , j2g3. , j2g4. , j2g5. , j2gx. , j2gy. , j2gz. , buB.. , buB1. , buBz. , bu6.. , bu61. , bu62. , bu8.. , bu81. , bu82. , bu8y. , bu8z. |
| Migraine |  | Four or more prescriptions for migraine medication issued per year. Medication read codes:  dl... , dl1.. , dl11. , dl12. , dl13. , dl14. , dl15. , dl16. , dl17. , dl18. , dl19. , dl1a. , dl1A. , dl1b. , dl1B. , dl1C. , dlE.. , dlE1. , dlE2. , dm... , dlC.. , dlC1. , dlCz. , dm1.. , dm11. , dm1z. , dl3.. , dl31. , dl32. , dl33. , dl3x. , dl3y. , dl3z. , dlB.. , dlB1. , dlB2. , dlD.. , dlD1. , dlD2. , dl2.. , dl23. , dl24. , dl2y. , dl2z. , dl21. , dl22. , dl25. , dl26. , dl27. , dlE.. , dlE1. , dq7.. , dq71. , dm2.. , dm21. , dm2z. , dl9.. , dl91. , dl92. , dm3.. , dm31. , dm32. , dm33. , dm3x. , dm3y. , dm3z. , dlA.. , dlA1. , dlA2. , dlA3. , dlA4. , dlA5. , dlA6. , dl5.. , dl51. , dl52. , dl53. , dl54. , dl55. , dl56. , dl57. , dl58. , dl59. , dl5A. , dl5B. , dl5C. , dl5D. , dl5E. , dl7.. , dl71. , dl72. , dl73. , dl74. , dl8.. , dl81. , dl82. , dl83. , dl84. , dl85. , dl86. , dl87. , dl88. |
| Painful Condition |  | Four or more prescriptions for painkillers issued per year. Medication read codes:  di... , diaN. , dibC. , dj... , dj23. , dj24. , dj26. , dl... , dl1.. , dl11. , dl12. , dl13. , dl14. , dl15. , dl16. , dl17. , dl18. , dl19. , dl1a. , dl1A. , dl1b. , dl1B. , dl1C. , dlE.. , dlE1. , dlE2. , dm... , j1... , di1.. , di11. , di12. , di15. , di16. , di17. , di18. , di1a. , di1c. , di1d. , di1f. , di1g. , di1h. , di1i. , di1k. , di1m. , di1n. , di1o. , di1r. , j11.. , j111. , j112. , dia4. , dia5. , dia8. , diab. , diaB. , diaE. , diaG. , diai. , diaO. , diaP. , diay. , diaz. , dicH. , diaf. , dlC.. , dlC1. , dlCz. , di1e. , di3.. , j12.. , j121. , j122. , j124. , j12x. , j12y. , j12z. , dj3.. , dj31. , dj32. , dj33. , dj34. , dj35. , dj3A. , dj3B. , dj3C. , dj3f. , dj3g. , dj3o. , dj3p. , dj3q. , dj3x. , dj3y. , dj3z. , j13.. , j131. , j13z. , dm1.. , dm11. , dm1z. , dj5.. , dj51. , dj52. , dj53. , dj54. , dj55. , dj56. , dj57. , dj58. , dj59. , dj5a. , dj6.. , dj61. , dj62. , dia7. , diad. , diae. , diaw. , dibh. , di4.. , j23.. , j231. , j232. , j233. , j234. , dj8.. , dj81. , dj82. , dj83. , dj84. , dj85. , dj86. , dj87. , dj88. , dj89. , dj8a. , dj8b. , dj8c. , dj8d. , dj8e. , dibE. , dibF. , dibH. , dibI. , dibJ. , dibK. , dibv. , dibw. , dibx. , dicb. , dicc. , dl3.. , dl31. , dl32. , dl33. , dl3x. , dl3y. , dl3z. , dlB.. , dlB1. , dlB2. , dlD.. , dlD1. , dlD2. , dl2.. , dl23. , dl24. , dl2y. , dl2z. , dl21. , dl22. , dl25. , dl26. , dl27. , o424. , o425. , o426. , o427. , o428. , o429. , o42a. , o42A. , o42b. , o42B. , o42c. , o42C. , o42d. , o42D. , o42e. , o42E. , o42f. , o42F. , o42g. , o42G. , o42h. , o42H. , o42i. , o42I. , o42j. , o42J. , o42k. , o42K. , o42l. , o42L. , o42m. , o42M. , o42n. , o42N. , o42o. , o42O. , o42p. , o42P. , o42q. , o42Q. , o42r. , o42R. , o42s. , o42S. , o42t. , o42T. , o42u. , o42U. , o42v. , o42V. , o42W. , o42X. , o42Y. , o42Z. , o4d.. , o4d1. , o4d2. , o4d3. , o4d4. , o4d5. , o4d6. , o4d7. , o4d8. , o4d9. , o4da. , o4dA. , o4db. , o4dB. , o4dc. , o4dC. , o4dd. , o4dD. , o4de. , o4dE. , o4df. , o4dF. , o4dg. , o4dh. , o4di. , o4dj. , o4dk. , o4dl. , o4dm. , o4dn. , o4do. , o4dp. , o4dq. , o4dr. , o4ds. , o4dt. , o4du. , o4dv. , o4dw. , o4dx. , o4dy. , o4dz. , dlE.. , dlE1. , djj.. , djj1. , djj2. , djj3. , djj4. , djj5. , djj6. , djj7. , djj8. , djj9. , djjA. , djjB. , djjC. , djjD. , djjE. , dq7.. , dq71. , djb.. , djb3. , djb4. , o44.. , o443. , o44z. , dm2.. , dm21. , dm2z. , dj1.. , dj11. , dj12. , dj13. , dj14. , dj15. , dj16. , dj17. , dj18. , dj19. , dj1a. , dj1A. , dj1B. , dj1C. , dj1D. , dj1e. , dj1E. , dj1f. , dj1F. , dj1g. , dj1G. , dj1h. , dj1H. , dj1i. , dj1I. , dj1j. , dj1J. , dj1k. , dj1K. , dj1l. , dj1L. , dj1m. , dj1M. , dj1n. , dj1N. , dj1o. , dj1O. , dj1p. , dj1P. , dj1q. , dj1Q. , dj1r. , dj1R. , dj1s. , dj1S. , dj1t. , dj1T. , dj1u. , dj1U. , dj1v. , dj1V. , dj1w. , dj1W. , dj1x. , dj1X. , dj1y. , dj1Y. , dj1z. , dj1Z. , dj2.. , dj21. , djy.. , djy1. , djy2. , djy3. , djy4. , djy5. , djy6. , djy7. , djy8. , djy9. , djyA. , djyB. , djyC. , djyD. , djyE. , djyF. , djyG. , djyH. , djyI. , djz.. , djz1. , djz2. , djz3. , djz4. , djz5. , djz6. , djz7. , djz8. , djz9. , djza. , djzA. , djzb. , djzB. , djzc. , djzC. , djzd. , djzD. , djze. , djzE. , djzf. , djzF. , djzg. , djzG. , djzh. , djzH. , djzi. , djzI. , djzj. , djzJ. , djzk. , djzK. , djzl. , djzL. , djzm. , djzM. , djzn. , djzo. , djzO. , djzp. , djzP. , djzq. , djzQ. , djzr. , djzR. , djzs. , djzS. , djzt. , djzT. , djzu. , djzU. , djzv. , djzV. , djzw. , djzW. , djzx. , djzX. , djzy. , djzY. , djzz. , djzZ. , o45.. , o451. , o452. , o453. , o454. , o455. , o456. , o457. , dia9. , dj1c. , dj1d. , dj25. , o458. , o473. , o47B. , di1j. , dj22. , dj7.. , dj71. , dj72. , dj73. , dj74. , dj75. , dj76. , dj77. , dj78. , dj79. , dj7A. , dj7B. , dj7C. , dj7D. , dj7E. , dj7F. , dj7G. , dA2.. , dA21. , dA22. , djd.. , djd1. , djd2. , djd3. , djdz. , o46.. , o461. , o462. , o46z. , dl9.. , dl91. , dl92. , di9.. , di91. , di92. , di9y. , di9z. , dj1b. , djk.. , djk1. , djk2. , djk3. , djk4. , djk5. , djk6. , djk7. , djk8. , djk9. , djkA. , djkB. , djke. , djkE. , djkf. , djkg. , djkh. , djkH. , djkI. , djkJ. , djkk. , djkK. , djkL. , djkM. , djkn. , djkN. , djko. , djkO. , djkp. , djkP. , djkq. , djkQ. , djkr. , djkR. , djks. , djkS. , djkt. , djkT. , djku. , djkU. , djkv. , djkV. , djkw. , djkW. , djkx. , djkX. , djky. , djkz. , djkC. , djkD. , djkF. , djkG. , djki. , djkj. , djkl. , djkm. , dje.. , o47.. , o471. , o472. , o474. , o475. , o476. , o477. , o478. , o479. , o47A. , o47y. , o47z. , o4b.. , o4b1. , o4b2. , di2.. , di21. , di22. , di23. , di24. , di25. , di26. , di27. , di28. , di29. , di2a. , di2A. , di2b. , di2B. , di2c. , di2C. , di2d. , di2e. , di2E. , di2F. , di2g. , di2h. , di2i. , di2I. , di2j. , di2J. , di2K. , di2l. , di2L. , di2m. , di2M. , di2n. , di2N. , di2o. , di2O. , di2p. , di2P. , di2q. , di2Q. , di2r. , di2R. , di2s. , di2S. , di2t. , di2T. , di2u. , di2U. , di2V. , di2w. , di2W. , di2x. , di2X. , di2y. , di2Y. , di2Z. , diaA. , diaC. , did.. , did1. , did2. , did3. , did4. , did5. , did7. , did8. , did9. , didA. , didB. , didC. , didD. , didE. , didF. , didG. , didH. , didu. , didv. , didw. , didx. , didy. , didz. , di2f. , di2G. , di2H. , di2v. , dia1. , dia2. , dia3. , dia6. , diaa. , diaD. , diaF. , diah. , diaH. , diaI. , diaJ. , diaK. , dial. , diaL. , diam. , diaM. , dian. , diao. , diap. , diaq. , diaQ. , diar. , diaR. , diaS. , diaT. , diau. , diaU. , diaV. , diaX. , diaY. , diaZ. , dib3. , dib5. , dib8. , diba. , dibb. , dibB. , dibD. , dibe. , dibf. , dibG. , dibj. , dibL. , dibM. , dibn. , dibN. , dibO. , dibp. , dibP. , dibQ. , dibR. , dibs. , dibS. , dibt. , dibT. , dibu. , dibU. , dibV. , dibW. , dibX. , dibY. , dibz. , dibZ. , dic1. , dic2. , dic3. , dic4. , dic5. , dic9. , dica. , dicA. , dicD. , dicE. , dicF. , dicG. , dicI. , dicJ. , dicK. , dicL. , dicM. , dicN. , dicQ. , dicR. , dicS. , dicT. , dicU. , dicv. , dicV. , dicW. , dicX. , dicy. , did6. , diaj. , dib2. , diby. , djf.. , djf1. , djf2. , djf3. , djf4. , djf5. , djf6. , djf7. , djf8. , djf9. , djfa. , djg.. , djg2. , djg3. , djg4. , djg6. , o48.. , o481. , o482. , o483. , o484. , djg5. , o485. , djh.. , djh1. , djhz. , dm3.. , dm31. , dm32. , dm33. , dm3x. , dm3y. , dm3z. , dlA.. , dlA1. , dlA2. , dlA3. , dlA4. , dlA5. , dlA6. , j14.. , j141. , j14z. , j15.. , j151. , j152. , dl5.. , dl51. , dl52. , dl53. , dl54. , dl55. , dl56. , dl57. , dl58. , dl59. , dl5A. , dl5B. , dl5C. , dl5D. , dl5E. , dl7.. , dl71. , dl72. , dl73. , dl74. , djB.. , djB1. , djB2. , djB3. , djB4. , djB5. , djB6. , djB7. , djBT. , djBU. , djBV. , djBW. , djBX. , djBY. , djBZ. , djA.. , djA1. , djA2. , djA3. , djA4. , djA5. , djA6. , djA7. , djA8. , djA9. , djAa. , djAb. , djAc. , djAd. , djAe. , djAf. , djAg. , djAh. , djAi. , djAj. , djAk. , djAl. , djAm. , djAn. , djAo. , dji.. , dji1. , dji2. , dji3. , dji4. , dji5. , dji6. , dji7. , dji8. , dji9. , djia. , djiA. , djib. , djiB. , djic. , djiC. , djid. , djiD. , djie. , djiE. , djif. , djiF. , djig. , djiG. , djih. , djiH. , djii. , djiI. , djij. , djiJ. , djik. , djiK. , djil. , djiL. , djim. , djiM. , djin. , djiN. , djio. , djiO. , djip. , djiP. , djiq. , djiQ. , djir. , djiR. , djis. , djiS. , djit. , djiT. , djiU. , djiv. , djiV. , djiw. , djiW. , djix. , djiX. , djiy. , djiY. , djiz. , djiZ. , dicO. , dicx. , dicY. , dicz. , die.. , dieA. , dieB. , diey. , diez. , dl8.. , dl81. , dl82. , dl83. , dl84. , dl85. , dl86. , dl87. , dl88.  The following medication read codes were aso included, unless the participant also had epilepsy:  dn3.. , dn31. , dn32. , dn33. , dn34. , dn35. , dn36. , dn37. , dn38. , dn39. , dn3a. , dn3A. , dn3b. , dn3B. , dn3c. , dn3C. , dn3d. , dn3D. , dn3e. , dn3E. , dn3f. , dn3F. , dn3G. , dn3H. , dn3I. , dn3J. , dn3K. , dn3v. , dn3w. , dn3x. , dn3y. , dn3z. , dnj.. , dnj1. , dnj2. , dnj3. , dnj4. , dnj5. , dnj6. , dnj7. , dnj8. , dnj9. , dnjA. , dnjx. , dnjy. , dnjz. , dnp.. , dnp1. , dnp2. , dnp3. , dnp4. , dnp5. , dnp6. , dnp7. , dnp8. , dnp9. , dnpr. , dnps. , dnpt. , dnpu. , dnpv. , dnpw. , dnpx. , dnpy. , dnpz. |

**Table S2. Long-term conditions considered and read code definitions used in Secure Anonymised Information Linkage Databank.** ^m^Mental health condition

| Method of Admission Code | Source | Method of Admission Description |
| --- | --- | --- |
| 2A | HES | Emergency: via A&E of another hospital provider |
| 2B | HES | Emergency: Transfer from another hospital provider |
| 2D | HES | Emergency: Other |
| 4 | SMR | Emergency: Deliberate Self injury or poisoning |
| 5 | SMR | Emergency: Road Traffic Accident |
| 6 | SMR | Emergency: Home Accident |
| 7 | SMR | Emergency: Other injury |
| 8 | SMR | Emergency: Other |
| 20 | SMR | Urgent Admission, no additional detail added |
| 21 | PEDW | Emergency: via A&E or dental casualty department |
| 22 | PEDW | Emergency: after GP request for immediate admission |
| 23 | PEDW | Emergency: Bed bureau |
| 24 | PEDW | Emergency: Consultant clinic of this or other provider |
| 25 | PEDW | Emergency: Domiciliary visit by Consultant |
| 27 | PEDW | Emergency: Via NHS Direct Services |
| 28 | PEDW | Emergency: Other means, incl. other A&E department |
| 29 | PEDW | Emergency: Other |
| 30 | SMR | Emergency Admission, no additional detail added |
| 31 | SMR | Patient Injury: Self Inflicted (Injury or Poisoning) |
| 32 | SMR | Patient Injury: Road Traffic Accident (RTA) |
| 33 | SMR | Patient Injury: Home Accident |
| 34 | SMR | Patient Injury: Accident at Work |
| 35 | SMR | Patient Injury: Other Injury, not elsewhere classified |
| 36 | SMR | Patient Non-Injury |
| 38 | SMR | Other Emergency Admission (including emergency transfers) |
| 39 | SMR | Emergency Admission, type not known |

**Table S3. Method of admission codes to identify emergency admissions.** HES, Hospital Episode Statistics, SMR, Scottish Morbidity Record, PEDW, Patient Episode Database for Wales, A&E Accident & Emergency, GP, General Practitioner, NHS National Health Service.

| SAIL | | Excluded  i.e. no kidney function available  N=990748 | Included  i.e. kidney function available  N=1620490 |
| --- | --- | --- | --- |
| Age (years) | Median (IQR) | 30 (20-45) | 50 (35-65) |
| Sex (%) | Female | 449800 (45.4) | 857305(54.7) |
| Ethnicity (%)  Missing values  1474539 (56.5%) | White | 392704 (86.1) | 645205 (94.8) |
|  | Black | 8665 (1.9) | 6125 (0.9) |
|  | Asian | 23261 (5.1) | 16334 (2.4) |
|  | Mixed | 6385 (1.4) | 3403 (0.5) |
|  | Other | 25085 (5.5) | 9528 (1.4) |
| WIMD score  Missing values  765231 (29.3%) | Median (IQR) | 17.8 (10.8-30.1) | 18.0 (10.8-29.8) |
| Smoking (%)  Missing values  1035418 (39.7%) | Never | 313633 (61.6) | 516300 (48.4) |
|  | Ex | 61097 (12.0) | 277351 (26.0) |
|  | Current | 134414 (26.4) | 274151 (25.7) |
| Body mass index  Missing values  2452490 (93.9%) | Median (IQR) | 24 (21-28) | 28 (24-33) |
| Systolic blood pressure  Missing values  1220472 (46.7%) | Median (IQR) | 120 (110-131) | 130 (120-142) |
| Long-term conditions | Median (IQR) | 0 (0-1) | 2 (0-3) |

**Table S4 Baseline characteristics in SAIL by study inclusion.** IQR, interquartile range, WIMD Welsh Index of Multiple Deprivation

| **UK Biobank** | | **No CKD, No LTCs n=159642 (34.0%)** | **No CKD, 1 LTC n=151149 (32.2%)** | **No CKD, 2 LTCs n=86731 (18.5%)** | **No CKD, 3 LTCs n=38556 (8.2%)** | **No CKD, 4 or more LTCs n=22494 (4.8%)** | **CKD, No additional LTCs n=1540 (0.3%)** | **CKD, 1 additional LTC n=2696 (0.6%)** | **CKD, 2 additional LTCs n=2808 (0.6%)** | **CKD, 3 or more additional LTCs n=3723 (0.8%)** | **p-value** |
| --- | --- | --- | --- | --- | --- | --- | --- | --- | --- | --- | --- |
| Age (years) | Median (IQR) | 54  (47 to 61) | 58  (50 to 63) | 60  (53 to 64) | 61  (55 to 65) | 62  (56 to 65) | 62  (57 to 66) | 64  (59 to 67) | 65  (61 to 67) | 65  (61 to 67) | <.001 |
| Sex (%) | Female | 85417 (53.5) | 81437 (53.9) | 47023 (54.2) | 21487 (55.7) | 13387 (59.5) | 947 (61.5) | 1448 (53.7) | 1453 (51.7) | 1898 (51.0) | <.001 |
|  | Male | 74225 (46.5) | 69712 (46.1) | 39708 (45.8) | 17069 (44.3) | 9107 (40.5) | 593 (38.5) | 1248 (46.3) | 1355 (48.3) | 1825 (49.0) |  |
| Ethnicity (%) | White | 149679 (94.2) | 142566 (94.8) | 82047 (95.0) | 36503 (95.2) | 21356 (95.5) | 1480 (96.5) | 2565 (95.7) | 2638 (94.5) | 3519 (95.0) | <.001 |
|  | Black | 2646 (1.7) | 2423 (1.6) | 1302 (1.5) | 538 (1.4) | 264 (1.2) | 7 (0.5) | 30 (1.1) | 41 (1.5) | 50 (1.3) |  |
|  | Asian | 3925 (2.5) | 3266 (2.2) | 1862 (2.2) | 805 (2.1) | 453 (2.0) | 19 (1.2) | 59 (2.2) | 69 (2.5) | 88 (2.4) |  |
|  | Mixed | 1028 (0.6) | 878 (0.6) | 486 (0.6) | 191 (0.5) | 122 (0.5) | 9 (0.6) | 7 (0.3) | 17 (0.6) | 15 (0.4) |  |
|  | Other | 1607 (1.0) | 1327 (0.9) | 669 (0.8) | 307 (0.8) | 175 (0.8) | 18 (1.2) | 18 (0.7) | 28 (1.0) | 32 (0.9) |  |
| Townsend Deprivation Score | Median (IQR) | -2.3 (-3.7 to 0.2) | -2.2 (-3.7 to 0.3) | -2.1 (-3.6 to 0.7) | -1.8 (-3.4 to 1.2) | -1.2 (-3.2 to 2.2) | -2.5 (-3.8 to -0.1) | -2.3 (-3.7 to 0.1) | -2.0 (-3.5 to 0.7) | -1.5 (-3.2 to 1.8) | <.001 |
| Smoking status (%) | Never | 94815 (59.7) | 83235 (55.3) | 44291 (51.3) | 18274 (47.7) | 9689 (43.4) | 893 (58.3) | 1425 (53.3) | 1374 (49.3) | 1569 (42.5) | <.001 |
|  | Previous | 47508 (29.9) | 51671 (34.3) | 33002 (38.3) | 15708 (41.0) | 9684 (43.4) | 531 (34.7) | 1045 (39.1) | 1181 (42.4) | 1801 (48.8) |  |
|  | Current | 16621 (10.5) | 15530 (10.3) | 8979 (10.4) | 4326 (11.3) | 2946 (13.2) | 108 (7.0) | 202 (7.6) | 230 (8.3) | 322 (8.7) |  |
| Body Mass Index (kg/m^2^) | Median (IQR) | 25.8 (23.5 to 28.5) | 26.6 (24.1 to 29.6) | 27.5 (24.8 to 30.8) | 28.3 (25.4 to 32.0) | 29.4 (26.1 to 33.6) | 26.7 (24.4 to 29.4) | 27.6 (25.0 to 30.7) | 28.5 (25.9 to 31.9) | 30.0 (26.8 to 33.8) | <.001 |
| Systolic Blood Pressure (mmHg) | Median (IQR) | 133 (122 to 145) | 137 (125 to 150) | 140 (128 to 153) | 140 (129 to 153) | 140 (128 to 153) | 137 (126 to 149) | 139 (127 to 152) | 140 (128 to 153) | 139 (126 to 153) | <.001 |
| Estimated Glomerular Filtration Rate  (ml/min/1.73m^2^) | Median (IQR) | 94.6 (85.8 to 101.9) | 93.0 (83.8 to 100.1) | 92.0 (82.2 to 98.7) | 91.4 (81.1 to 98.1) | 90.9 (79.8 to 97.7) | 56.0 (52.7 to 58.3) | 54.9 (49.7 to 57.8) | 53.7 (47.4 to 57.5) | 52.3 (45.1 to 56.8) | <.001 |
| Urine Albumin-Creatinine Ratio (mg/mmol) | Median (IQR) | 0.0 (0.0 to 0.4) | 0.0 (0.0 to 0.6) | 0.0 (0.0 to 0.7) | 0.0 (0.0 to 0.9) | 0.0 (0.0 to 1.1) | 0.0 (0.0 to 0.7) | 0.0 (0.0 to 1.4) | 0.5 (0.0 to 2.2) | 0.7 (0.0 to 2.9) | <.001 |

**Table S5. Baseline characteristics by chronic kidney disease (CKD) status and number of long-term conditions (LTCs) for UK Biobank.** IQR, interquartile range

| **SAIL** | | **No CKD, No LTCs n=442601 (27.3%)** | **No CKD, 1 LTC n=375376 (23.2%)** | **No CKD, 2 LTCs**  **n=290614 (17.9%)** | **No CKD, 3 LTCs n=169272 (10.4%)** | **No CKD, 4 or more LTCs n=169239 (10.4%)** | **CKD, No additional LTCs n=4311 (0.3%)** | **CKD, 1 additional LTC n=17460 (1.1%)** | **CKD, 2 additional LTCs n=29539 (1.8%)** | **CKD, 3 or more additional LTCs n=122078 (7.5%)** | **P-value** |
| --- | --- | --- | --- | --- | --- | --- | --- | --- | --- | --- | --- |
| Age (years) | Median  (IQR) | 43  (29 to 55) | 50  (37 to 62) | 52  (39 to 64) | 56  (43 to 67) | 62  (50 to 72) | 76  (69 to 84) | 77  (70 to 84) | 78  (71 to 84) | 79  (72 to 85) | <.001 |
| Sex (%) | Female | 218064 (49.3) | 199814 (53.2) | 169345 (58.3) | 100293 (59.2) | 101209 (59.8) | 2374 (55.1) | 9577 (54.9) | 16348 (55.3) | 70483 (57.7) | <.001 |
|  | Male | 224537 (50.7) | 175562 (46.8) | 121269 (41.7) | 68979 (40.8) | 68030 (40.2) | 1937 (44.9) | 7883 (45.1) | 13191 (44.7) | 51595 (42.3) |  |
| Ethnicity (%) | White | 174878 (89.6) | 158847 (94.9) | 130368 (96.8) | 78060 (97.7) | 80171 (98.3) | 1465 (98.0) | 6480 (98.4) | 11441 (98.5) | 52038 (98.7) | <.001 |
|  | Black | 3355 (1.7) | 1412 (0.8) | 723 (0.5) | 303 (0.4) | 239 (0.3) | <15 | <15 | 25 (0.2) | 88 (0.2) |  |
|  | Asian | 9195 (4.7) | 3951 (2.4) | 1925 (1.4) | 797 (1.0) | 631 (0.8) | 18 (1.2) | 50 (0.8) | 97 (0.8) | 370 (0.7) |  |
|  | Mixed | 1835 (0.9) | 973 (0.6) | 593 (0.4) | 233 (0.3) | 172 (0.2) | <15 | <15 | <15 | 77 (0.1) |  |
|  | Other | 5961 (3.1) | 2282 (1.4) | 1033 (0.8) | 467 (0.6) | 318 (0.4) | <15 | 32 (0.5) | 42 (0.4) | 142 (0.3) |  |
| Welsh Index of Multiple Deprivation Rank | Median (IQR) | 17.1 (10.4 to 27.7) | 17.3 (10.5 to 28.4) | 18.4 (11.0 to 30.6) | 19.3 (11.4 to 31.9) | 20.4 (12.2 to 34.1) | 16.8 (10.5 to 26.9) | 16.8 (10.5 to 26.7) | 17.1 (10.8 to 27.0) | 18.0 (11.2 to 29.0) | <.001 |
| Smoking Status (%) | Never | 169456 (57.5) | 139770 (51.1) | 99381 (44.8) | 55789 (41.4) | 52332 (36.9) | 1601 (54.7) | 7139 (54.0) | 11805 (51.1) | 44587 (45.5) | <.001 |
|  | Previous | 57520 (19.5) | 67446 (24.6) | 61184 (27.6) | 40334 (29.9) | 47424 (33.4) | 988  (33.7) | 4649 (35.2) | 8992 (38.9) | 43561 (44.4) |  |
|  | Current | 67861 (23.0) | 66492 (24.3) | 61287 (27.6) | 38711 (28.7) | 42194 (29.7) | 340  (11.6) | 1426 (10.8) | 2311 (10.0) | 9885 (10.1) |  |
| Body Mass Index | Median (IQR) | 27  (23 to 31) | 28  (24 to 32) | 29  (25 to 33) | 29  (25 to 34) | 30  (25 to 34) | 27  (24 to 30) | 28  (24 to 31) | 28  (25 to 32) | 29  (25 to 32) | <.001 |
| Systolic Blood Pressure (mmHg) | Median (IQR) | 128 (118 to 140) | 130 (120 to 142) | 130 (120 to 142) | 131 (120 to 142) | 132 (120 to 143) | 136 (124 to 144) | 136 (126 to 146) | 136 (125 to 145) | 133 (120 to 143) | <.001 |
| Estimated Glomerular Filtration Rate  (ml/min/1.73m2) | Median (IQR) | 102.7  (90.7 to 115.4) | 97.3  (85.9 to 109.2) | 95.9  (84.4 to 107.7) | 93.3  (82.3 to 104.7) | 89.6  (79.2 to 100.3) | 52.4  (45.7 to 56.6) | 52.2  (44.9 to 56.6) | 51.9  (44.0 to 56.5) | 50.8  (42.2 to 56.1) | <.001 |
| Albumin-Creatinine Ratio (mg/mmol) | Median (IQR) | 0.8 (0.5 to 1.7) | 0.8 (0.5 to 1.7) | 0.9 (0.5 to 2.0) | 0.9 (0.5 to 2.1) | 1.1 (0.6 to 2.6) | 1.1 (0.6 to 3.3) | 1.3 (0.7 to 3.6) | 1.5 (0.7 to 4.7) | 1.9 (0.8 to 6.2) | <.001 |

**Table S6. Baseline characteristics by chronic kidney disease (CKD) status and number of long-term conditions (LTCs) for SAIL.** IQR, interquartile range

| LTCs | UK Biobank | | | | | | | | | | SAIL | | | | | | | | | |
| --- | --- | --- | --- | --- | --- | --- | --- | --- | --- | --- | --- | --- | --- | --- | --- | --- | --- | --- | --- | --- |
|  | No CKD | | | | | CKD | | | | | No CKD | | | | | CKD | | | | |
|  | Events per 100 person years | Unadjusted | | Adjusted* | | Events per 100 person years | Unadjusted | | Adjusted* | | Events per 100 person years | Unadjusted | | Adjusted* | | Events per 100 person years | Unadjusted* | | Adjusted* | |
|  |  | Rate Ratio | P-value | Rate Ratio | P-value |  | Rate ratio | P-value | Rate ratio | P-value |  | Rate Ratio | P-value | Rate Ratio | P-value |  | Rate Ratio | P-value | Rate Ratio | P-value |
| 0 | 2.45 | 1.00 (ref.) |  | 1.00 (ref.) |  |  |  |  |  |  | 3.02 | 1.00 (ref.) |  | 1.00 (ref.) |  |  |  |  |  |  |
| 1 | 2.96 | 1.47 (1.45-1.49) | <.001 | 1.47 (1.45-1.49) | <.001 | 2.76 | 1.34 (1.22-1.47) | <.001 | 1.36 (1.23-1.52) | <.001 | 3.36 | 1.52 (1.51-1.54) | <.001 | 1.27 (1.25-1.29) | <.001 | 6.45 | 3.28 (3.12-3.46) | <.001 | 3.34 (3.11-3.60) | <.001 |
| 2 | 3.50 | 2.08 (2.05-2.11) | <.001 | 2.09 (2.05-2.12) | <.001 | 3.61 | 2.59 (2.42-2.77) | <.001 | 2.75 (2.55-2.96) | <.001 | 3.88 | 2.15 (2.13-2.17) | <.001 | 1.70 (1.67-1.72) | <.001 | 6.50 | 3.71 (3.62-3.81) | <.001 | 3.54 (3.42-3.67) | <.001 |
| 3 | 4.09 | 3.02 (2.96-3.08) | <.001 | 3.02 (2.96-3.08) | <.001 | 4.49 | 3.90 (3.69-4.12) | <.001 | 4.43 (4.14-4.74) | <.001 | 4.38 | 2.99 (2.95-3.03) | <.001 | 2.23 (2.20-2.27) | <.001 | 7.23 | 4.46 (4.38-4.54) | <.001 | 4.57 (4.45-4.70) | <.001 |
| 4 or more | 4.81 | 4.78 (4.67, 4.89) | <.001 | 4.95 (4.82-5.06) | <.001 | 5.23 | 6.40 (6.13-6.69) | <.001 | 7.83 (7.42-8.25) | <.001 | 5.38 | 4.96 (4.91-5.02) | <.001 | 3.77 (3.71-3.82) | <.001 | 10.16 | 7.54 (7.46-7.62) | <.001 | 9.92 (9.75-10.09) | <.001 |

**Table S7. Hospitalisation Events by Chronic Kidney Disease (CKD) status and number of long-term conditions (LTCs).** *Adjusted for age, sex, deprivation status and smoking status

| **Body System** | **Hospitalisation Events (%)** | | | | | |
| --- | --- | --- | --- | --- | --- | --- |
|  | **UK Biobank** | | | **SAIL** | | |
|  | **No CKD** | **CKD** | **P-value** | **No CKD** | **CKD** | **P-value** |
| Circulatory | 98 522 (18.1) | 6 696 (22.4) | <.001 | 211 594 (15.5) | 116 788 (23.7) | <.001 |
| Metabolic | 5 535 (1.0) | 903 (3.0) |  | 164 486 (12.0) | 119 920 (24.3) |  |
| Gastrointestinal | 49 531 (9.1) | 2 685 (9.0) |  | 88 582 (6.5) | 22 401 (4.5) |  |
| Respiratory | 29 655 (5.5) | 1 988 (6.7) |  | 74 240 (5.4) | 14 600 (3.0) |  |
| Injuries | 64 076 (11.8) | 2 794 (9.4) |  | 37 103 (2.7) | 4 118 (0.8) |  |
| Genitourinary | 19 593 (3.6) | 2 160 (7.2) |  | 30 344 (2.2) | 9 399 (1.9) |  |
| Neoplasms | 15 920 (2.9) | 834 (2.8) |  | 90 045 (6.6) | 31 216 (6.3) |  |
| Musculoskeletal | 23 878 (4.4) | 1 259 (4.2) |  | 27 934 (2.0) | 6 757 (1.4) |  |
| Neurological | 18 916 (3.5) | 706 (2.4) |  | 54 356 (4.0) | 12 551 (2.5) |  |
| Infections | 5 701 (1.0) | 434 (1.5) |  | 25 556 (1.9) | 9 114 (1.8) |  |
| Dermatological | 13 063 (2.4) | 713 (2.4) |  | 20 016 (1.5) | 4 697 (1.0) |  |
| Haematological | 2 910 (0.5) | 338 (1.1) |  | 34 224 (2.5) | 23 501 (4.8) |  |
| Congenital Abnormalities | 287 (0.1) | 74 (0.2) |  | 805 (0.1) | 98 (0.0) |  |
| Other | 71 022 (13.1) | 1 760 (5.9) |  | 248 869 (18.2) | 36 497 (7.4) |  |
| Missing | 125 372 (23.0) | 6 487 (21.7) |  | 258 009 (18.9) | 81 923 (16.6) |  |

**Table S8. Causes of Hospitalisation.** CKD, chronic kidney disease

| LTCs | No CKD | | | CKD | | |
| --- | --- | --- | --- | --- | --- | --- |
|  | Events per 100 participant years | Unadjusted | Adjusted* | Events per 100 participant years | Unadjusted | Adjusted* |
|  |  | Rate Ratio | Rate Ratio |  | Rate Ratio | Rate Ratio |
| 0 | 2.45 | 1.0 (re.) | 1.0 (ref.) |  |  |  |
| 1 | 2.96 | 1.46 (1.44, 1.48) | 1.47 (1.45, 1.49) | 2.85 | 1.40 (1.28, 1.53) | 1.41 (1.29, 1.55) |
| 2 | 3.50 | 2.07 (2.04, 2.11) | 2.08 (2.05, 2.12) | 3.62 | 2.61 (2.45, 2.77) | 2.75 (2.56, 2.94) |
| 3 | 4.08 | 3.00 (2.95, 3.06) | 3.00 (2.94, 3.07) | 4.50 | 3.89 (3.7, 4.1) | 4.38 (4.12, 4.67) |
| 4 or more | 4.80 | 4.76 (4.65, 4.87) | 4.92 (4.79, 5.06) | 5.27 | 6.4 (6.14, 6.67) | 7.76 (7.38, 8.16) |

**Table S9. UK Biobank Sensitivity analysis. Analysis categorising participants with albuminuria as Chronic Kidney Disease (CKD). Hospitalisation Events by CKD status and number of long-term conditions (LTCs).** *Adjusted for age, sex, Welsh Index of Multiple Deprivation and smoking status. P-values <.001 for all comparisons

| LTCs | No CKD | | | CKD | | |
| --- | --- | --- | --- | --- | --- | --- |
|  | Events per 100 participant years | Unadjusted | Adjusted* | Events per 100 participant years | Unadjusted | Adjusted* |
|  |  | Rate Ratio | Rate Ratio |  | Rate Ratio | Rate Ratio |
| 0 | 1.57 | 1.0 (ref.) | 1.0 (ref.) |  |  |  |
| 1 | 2.08 | 1.75 (1.73-1.77) | 1.43 (1.42-1.45) | 6.45 | 4.88 (4.64-5.14) | 7.06 (6.63-7.52) |
| 2 | 2.47 | 2.43 (2.40-2.46) | 1.89 (1.87-1.91) | 6.50 | 5.52 (5.39-5.66) | 7.42 (7.19-7.66) |
| 3 | 2.95 | 3.36 (3.32-3.39) | 2.53 (2.51-2.56) | 7.23 | 6.63 (6.51-6.75) | 9.62 (9.39-9.86) |
| 4 or more | 3.97 | 5.79 (5.74-5.85) | 4.61 (4.56-4.66) | 10.16 | 11.22 (11.11-11.32) | 21.20 (20.87-21.50) |

**Table S10. SAIL Sensitivity analysis 1. Analysis including participants without biochemistry categorised as no Chronic Kidney Disease (CKD). Hospitalisation Events by CKD status and number of long-term conditions (LTCs).** * Adjusted for age, sex, Welsh Index of Multiple Deprivation and smoking status. P-values <.001 for all comparisons

| LTCs | No CKD | CKD |
| --- | --- | --- |
|  | Hazard Ratio* | Hazard Ratio* |
| 0 | 1.0 (ref.) |  |
| 1 | 1.35 (1.33-1.36) | 3.56 (3.42-3.76) |
| 2 | 1.62 (1.61-1.64) | 4.00 (3.91-4.10) |
| 3 | 1.92 (1.91-1.94) | 4.87 (4.78-4.96) |
| 4 or more | 2.54 (2.52-2.57) | 8.19 (8.10-8.29) |

**Table S11. SAIL Sensitivity analysis 2. Analysis using chronic kidney disease (CKD) diagnosis as a time-varying covariate. Hospitalisation Events by CKD status and number of long-term conditions (LTCs).** * Adjusted for age, sex, Welsh Index of Multiple Deprivation and smoking status. P-values <.001 for all comparisons

| LTCs | No CKD | CKD |
| --- | --- | --- |
|  | Rate Ratio* | Rate Ratio* |
| 0 | 1.00 (ref.) |  |
| 1 | 1.27 (1.25-1.29) | 3.34 (3.09-3.61) |
| 2 | 1.70 (1.67-1.72) | 3.54 (3.41-3.68) |
| 3 | 2.23 (2.19-2.27) | 4.57 (4.43-4.72) |
| 4 or more | 3.77 (3.70-3.83) | 9.92 (9.71-10.13) |

**Table S12. SAIL Sensitivity analysis 3. Complete case analysis: Hospitalisation Events by Chronic Kidney Disease (CKD) status and number of long-term conditions (LTCs).** * Adjusted for age, sex, Welsh Index of Multiple Deprivation and smoking status. P-values <.001 for all comparisons
